# Coexisting Topological Hinges and 1D Rashba States in Bi_0.97_Sb_0.03_ Revealed by the Josephson Effect

**DOI:** 10.1021/acsnano.6c01686

**Published:** 2026-07-29

**Authors:** Biplab Bhattacharyya, Stijn R. de Wit, Zhen Wu, Yingkai Huang, Mark S. Golden, Alexander Brinkman, Chuan Li

**Affiliations:** † MESA+ Institute, 3230 University of Twente, Enschede 7500 AE, The Netherlands; ‡ Van der Waals−Zeeman Institute, IoP, 84709 University of Amsterdam, Amsterdam 1090 GL, The Netherlands

**Keywords:** higher-order topological states, hinge modes, topological Dirac semimetal, Josephson junction, ballistic transport, Rashba modes

## Abstract

Second-order topological insulating (SOTI) states in three-dimensional materials are helical one-dimensional hinge states. Inducing superconductivity in these states leads to gapless bound states, characterized by the 4π-periodic current-phase relation. Here, we provide evidence of the topologically protected hinge states in Dirac semimetal Bi_0.97_Sb_0.03_ nanoflakes by an unconventional interference pattern in a magnetic field and the 4π-periodic supercurrent carried by these states via the suppressed first and third Shapiro steps. Tight-binding simulations confirm the presence of multiple hinge modes, supporting our interpretation of Bi_0.97_Sb_0.03_ as a prototypical designable SOTI platform. The quantum confinement effect is identified by a quasi-one-dimensional bulk transport, and the confined Rashba states are responsible for the broadened hinge states.

## Graphical abstract

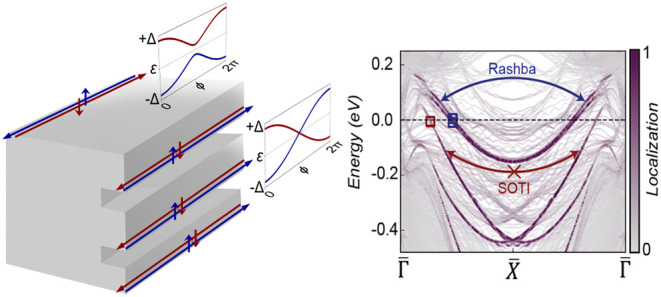

## 1 Introduction

Inducing superconductivity into topological materials in hybrid devices holds great interest for building topological quantum computing.^1,2^ The topological protection of the surface states is manifested by the spin-momentum locking and the suppression of backscattering. When superconductivity is induced in the helical states via the Josephson effect, parity-protected gapless bound states will form, namely, the Majorana bound states. Although such topological states can be found in different dimensions, their one-dimensional form provides the most significant physics due to its restricted spin degree of freedom. The 1D helical states can be realized as the edge state of a 2D topological insulator (TI) system (namely the quantum spin Hall states) when the time-reversal symmetry and inversion symmetry are preserved^3,4^ or the recently discovered hinge states in 3D second-order TIs (SOTIs) which are protected by crystalline symmetries in three dimensions.^5–10^ Higher dimensionality warrants the latter with stronger robustness against material defects and perturbations.

Despite the advances in the field, the formation and stability of hinge modes in response to local defects or geometric features of the material remain subjects of ongoing debate. An investigation of these aspects will yield essential insights into the nature of topological states and advance the development of quantum devices based on these novel topological phases. Theoretical predictions have been made for switching on and off the hinge states, via *e*–*e* interactions and Rashba spin–orbit coupling^11^ or via quantum confinement.^12^ However, so far, the experimental evidence is still lacking. On the other hand, the potential to host Majorana zero modes (MZMs) in helical hinge states via proximity-induced superconductivity has attracted considerable interest.^13–15^ In a Josephson junction configuration, the MZMs give rise to a 4π-periodic contribution to the supercurrent as a function of the phase difference between the two superconducting leads^14^ (Figure 1a). This can be measured as doubled Shapiro steps as a response to the high-frequency signal.^16–19^ Recently, a switching current measurement in a bismuth nanoring demonstrates the parity protection of the helical hinge states.^20^

**Figure 1:**
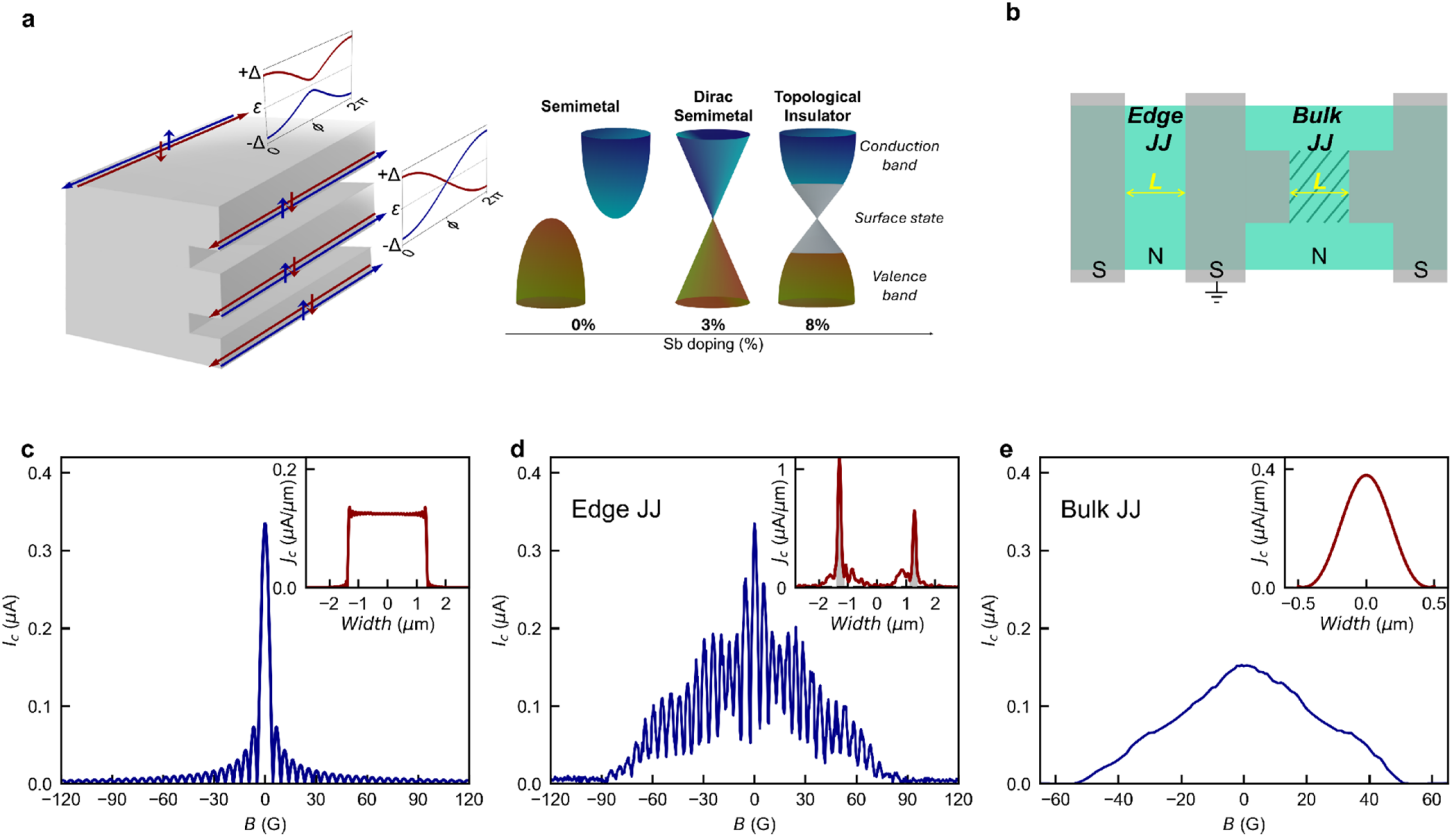
Evidence of edge supercurrent in Bi_0.97_Sb_0.03_ Josephson junctions. **a**, *left*: Schematic representation of multiple helical 1D hinge modes along the step edges in a 3D second-order topological insulator (SOTI). The hinge modes can host exotic gapless 4π-periodic Andreev bound states. In contrast, the bulk states host 2π-periodic Andreev bound states, with a gap opening due to the lack of topological protection. *right*: Classification of various material phases of Bi_1–*x*_Sb*_x_* alloy based on Sb doping percentage. **b**, Schematic top-view drawing of sample F1: the bulk JJ (on the top surface, avoiding edges) and the edge JJ (spanning the full width of the flake, including edges). The active junction area in the bulk JJ is indicated by diagonal hatching. *S*: superconductor (Nb), *N*: normal region (Bi_0.97_Sb_0.03_). Both junctions have length *L* = 800 nm. **c**, Simulated Fraunhofer pattern (FP) for a JJ with a homogeneous current distribution. The inset shows the calculated *J_c_* (*x*) profile without edge enhancement. **d, e**, Comparison of *I_c_* (*B*) interference patterns for bulk *W* = 1 μm and edge *W* = 2.7 μm JJs on flake F1. The edge JJ (**d**) exhibits SQUID-like oscillations, while the bulk JJ (**e**) shows a monotonically decaying non-oscillating *I_c_* (*B*) pattern. The inset in **d** depicts the extracted *J_c_* (*x*) for the edge JJ, with strongly enhanced edge currents (gray shaded area under the edge peaks) and negligible bulk contribution. The inset in **e** depicts a ″Gaussian-like″ *J_c_* (*x*) peaked at the center, suggesting (quasi-) 1D ballistic transport in confined geometries.

While the topological nature of the hinge states is revealed by the 4π-periodic supercurrent, its spatial distribution—localization on the edge—can be detected by the superconducting quantum interference (SQI) of the critical current *I*_*c*_(*B*). An enhanced supercurrent density *J*_*c*_ at the edge of the flake results in an interference pattern similar to the superconducting quantum interference device (SQUID), different from the standard Fraunhofer pattern (FP) obtained for the homogeneous current distribution^21,22^ (Figure 1c). Such an unusual feature was reported previously in various systems that were also identified with topological edge states, including the 2D TI^19,23^ and 3D SOTIs.^24–32^

In this work, we focus on Bi_1–*x*_Sb_*x*_—a large-scale compatible, air-stable, and free of volatile or toxic elements system. Depending on the doping, *x*, its phase ranges from semimetal to topological insulator (Figure 1a).^33,34^ We show that when the system is tuned to the Dirac semimetal phase at 3% Sb doping, it exhibits robust hinge states revealed by enhanced edge supercurrent. We compare the results to a tight-binding model and find out that multiple hinges are formed due to the natural cleave edges of the Bi_1–*x*_Sb_*x*_ crystal. Each hinge can host a SOTI channel. The high-frequency response not only shows a fractional Shapiro steps up to the second order but also demonstrates its robustness in temperature and, for the first time, a strong correlation with the existence of the hinge modes, together indicating the topological nature of the hinge modes. Strikingly, we also find a series of 1D Rashba states strongly localized at the edges. They turn out to be responsible for the broadening of the commonly observed edge current.

## 2 Results and Discussion

### 2.1 Bulk-Edge Junctions: Quasi-1D Transport

Bi_0.97_Sb_0.03_ flakes are exfoliated along the (111) plane. We fabricated 12 Josephson junctions (JJs) with lengths *L* ranging from 300 to 1000 nm and BiSb flake thicknesses *t* varying between 50 and 250 nm. Since the 4π-periodic Josephson supercurrent in long-ballistic junctions can exceed the conventional 2π-periodic supercurrent,^35^ and diffusive contributions are suppressed in this regime, all JJs were designed to be in the (medium-to-)­long junction regime. Here, the junction length *L* exceeds the superconducting coherence length ξ_*s*_, which enhances the ratio of the 4π-periodic contribution to the total Josephson supercurrent. In the main text, we focus on the results of the junctions on flakes F1 and F2. All the measurements were carried out at the base temperature of 70 mK, unless indicated otherwise. Details for the other devices are provided in the Supporting Information.

To first demonstrate the strongly enhanced edge supercurrent, we fabricated two junctions on a same Bi_0.97_Sb_0.03_ flake F1: one in which the superconducting leads cross the entire junction width (edge JJ), and another where the leads are confined near the center of the junction, leaving the edges largely uncovered (bulk JJ). Both junctions have identical lengths of *L* = 800 nm. A schematic of the junction geometries is shown in Figure 1b. The measured *I*_*c*_(*B*) patterns, shown in Figure 1d and 1e for the edge and bulk JJ, respectively, reveal two interesting features. First, the edge JJ exhibits a pronounced SQUID-like interference pattern indicative of strong edge supercurrent (Figure 1d). The real-space supercurrent density *J*_*c*_(*x*), calculated from the measured *I*_*c*_(*B*) using the Dynes and Fulton method^36^ (see Supplementary Section S3 for details), shows a dominant edge contribution. Second, for a homogeneous bulk current distribution, the expected *I*_*c*_(*B*) should follow a standard Fraunhofer pattern, *I*_*c*_ = *I*_*c*,max_ |sinc(ϕ/ϕ_0_)|, where ϕ = *BL*_eff_ *W* is the magnetic flux through the junction and ϕ_0_ is the flux quantum. However, the bulk JJ instead exhibits a nearly non-oscillatory *I*_*c*_(*B*) response, rather than a Fraunhofer-like modulation. This behavior indicates quasi-1D transport (Figure 1e).

The enhanced edge supercurrent can be related to the SOTI states forming at the hinges. However, from the *J*_*c*_(*x*) profile, we estimate the *I*_*c*_ at two sides to be *I*_*c*,*L*_ = 130 nA and *I*_*c*,*R*_ = 80 nA, respectively, exceeding the theoretical maximum for a single helical mode for SOTIs,^29,30^ suggesting that there are multiple edge channels contributing to the supercurrent. The atypical shape of the bulk JJ’s interference pattern may stem from the bulk quantization effects in the relatively small Bi_1–*x*_Sb_*x*_ flake (*W* ∼ 2300 nm).^37,38^ Similar phenomenon of a few quasi-1D ballistic modes has been observed in quantum point contact devices of InAs-based heterostructure and graphene.^39,40^ We attribute this unusually large-scale quantum confinement effect to the extremely small L-pocket in Bi_0.97_Sb_0.03_. We will discuss these two points further in this report.

### 2.2 Ballistic Junction – *I_c_* (*T*)

To further understand the transport properties of the junctions, we investigate the temperature dependence and the length dependence of the junctions. We present the results of the junctions with various lengths on another flake (F2). The inset of Figure 2a shows a false-color scanning electron microscopy (SEM) image of the flake F2 (width *W* = 1.4 μm, thickness *t* = 250 nm), which hosts four JJs with lengths of 600, 800, 900, and 1000 nm. Figure 2a shows the magnetic field dependence of the critical current *I*_*c*_(*B*) for the 600 nm long junction. A pronounced SQUID-like modulation is observed, with all lobes exhibiting nearly equal widths of Δ*B* ≃ 18 G. This corresponds to the magnetic flux quantum threading the effective junction area (*W* · *L*_eff_), where *L*_eff_ ≈ 900 nm accounts for flux-focusing effects due to the Nb electrodes. The calculated supercurrent density distribution *J*_*c*_(*x*), shown in Figure 2b, reveals a pronounced enhancement of *J*_*c*_ at both edges of the junction. Contrary to the intuitive expectation of a spatially uniform bulk supercurrent, the edge supercurrent in Figure 2b smoothly decays into the bulk. Using our superconducting quantum interference (SQI) model, we attribute this spatial profile to different suppression rates for the bulk and edge current by the magnetic field. This leads to more robust oscillations at a higher field. Furthermore, a phenomenological smoothing effect—arising from finite coherence within the junction—modifies the extracted profile, meaning that the calculated *J*_*c*_(*x*) profile does not precisely reflect the zero-field current distribution (see details in Supplementary Section S1). Unlike previous reports,^23,28–30^ the edge current profile in our data cannot be fitted by a simple Gaussian function. To consistently quantify the edge contribution, we develop a systematic procedure to determine, what we dub as the “optimal width” the spatial extent of the edge states that yields the best agreement between the extracted and actual supercurrent density. Details of the extraction and optimization procedure are provided in Supplementary Section S1. In Figure 2b, the shaded gray regions indicate the edge supercurrent contributions for the optimal width, while the dashed line denotes the residual bulk supercurrent. For the F2_600 junction, the extracted edge width is approximately 250 nm on each side, yielding left and right edge supercurrents of *I*_*c*,*L*_ = 1.19 μA and *I*_*c*,*R*_ = 0.93 μA, respectively, and a bulk supercurrent of *I*_*c*,bulk_ = 1.64 μA.

**Figure 2:**
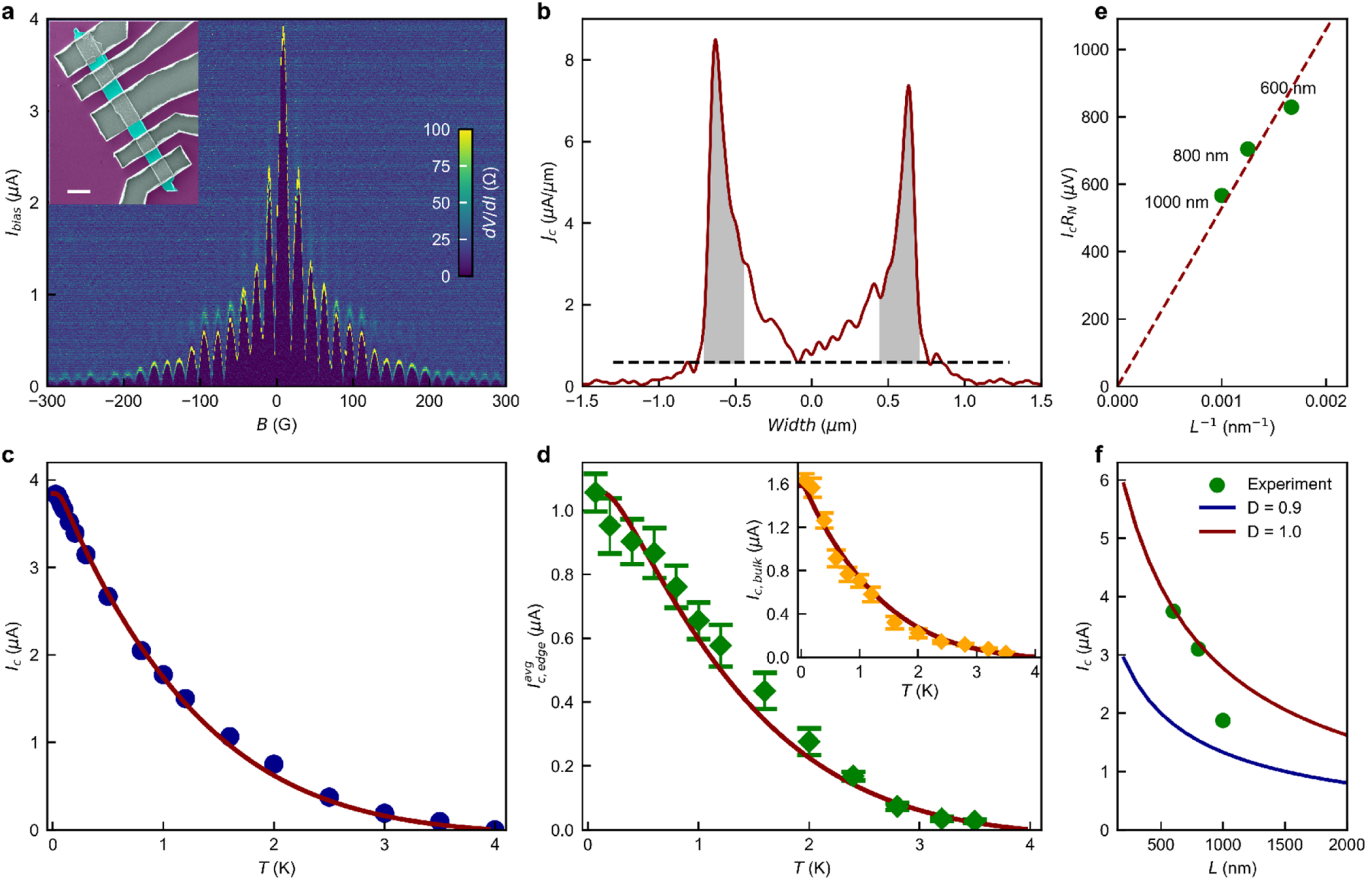
Ballistic transport in Bi_0.97_Sb_0.03_ junctions. **a**, Quantum interference pattern recorded at 70 mK for the F2_600 junction, displaying SQUID-like oscillations in *I_c_* (*B*). The colormap represents the differential resistance *dV*/*dI*. Inset: SEM image of the flake F2 with four junctions of lengths 600, 1000, 900, and 800 nm (from top to bottom). Scale bar: 2 μm. Color scheme matches Figure 1b. **b**, Extracted *J_c_* distribution across the junction width, calculated from **a**. Contribution from hinge modes is shown by the gray-shaded area. Residual bulk supercurrent is indicated by the black dashed line. **c**, Temperature dependence of *I_c_* for device F2_600. Measured data points (dark blue) can be fitted with the Eilenberger model (solid dark red) for ballistic junctions with near-unity interface transparency. **d**, Temperature dependence of the extracted *I_c_* for the edge modes (green diamonds) from the *J*_c_ distribution using the method described in Supplementary Section S2. The inset shows the same for the extracted bulk *I_c_* (orange diamonds). Both edge and bulk *I_c_* (*T*) can be fitted with the Eilenberger model (solid dark red). The error bars are estimated from the standard deviation between experimentally and numerically calculated *J_c_* distribution as described in Supplementary Section S1. **e**, *I_c_R_N_* scaling with the inverse of junction length for different devices on flake F2 at 70 mK. The red dashed line highlights the linear trend. **f**, *I_c_* scaling with junction length for the same devices as in **e**, where experimental values (green dots) lie within the Eilenberger framework, with upper (solid dark red) and lower (solid dark blue) bounds set by the interface transparency D = 0.9 and 1.0, respectively.

The temperature dependence of the critical current *I*_*c*_(*T*) is shown in Figure 2c (dots). The *I*_*c*_ exhibits an almost nonsaturating increase at low temperatures, which is indicative of a highly transparent interface.^41–44^ Previous studies on Bi_0.97_Sb_0.03_ have shown that this material hosts high-mobility carriers with exceptionally long mean-free paths,^45^ suggesting that our junctions are in the ballistic regime. To analyze the data quantitatively, we fit the measured *I*_*c*_(*T*) using the Eilenberger formalism developed by Galaktionov and Zaikin.^46^ The fit (solid line in Figure 2c) corresponds to a junction with *L* = 600 nm and yields the following parameters: interface transparency *D* = 0.9995 (assumed equal for both left and right interfaces) and normal-state resistance *R*_*N*_ = 208 Ω, corresponding to 62 modes. The superconducting coherence length is estimated to be ξ_*s*_ = 240 nm, taking *T*_*c*_ ∼ 4 K from the measurement and *v*_*F*_ ∼ 8 × 10^5^ m/s as an effective Fermi velocity. In this configuration, the superconducting Nb leads (with a *T*_*c*_ of ∼ 9 K) first proximitize the Bi_1–*x*_Sb_*x*_ underneath. These proximitized Bi_1–*x*_Sb_*x*_ regions act as superconducting leads for the lateral junction, with a critical temperature that is lower than that of Nb. There is no physical barrier between the proximitized and non-proximitized Bi_1–*x*_Sb_*x*_. As the carrier density in Bi_0.97_Sb_0.03_ is sufficiently high, the work function mismatch across this interface is negligible, providing a near-unity transparency of the lateral interfaces. In the case of a topological edge channel, the unit transparency is further safeguarded by the topologically prohibited backscattering. Similar high-transparency proximity effects in Bi_1–*x*_Sb_*x*_ have been reported in earlier studies.^45,47^ The fitted *R*_*N*_ value is notably higher than the experimentally measured *R*_*N*_ = 20 Ω. This is due to the presence of multiple transport channels: while all channels contribute to the normal-state resistance, only a subset of them may carry supercurrent. The overall fit supports the conclusion that the junction is in the intermediately long-ballistic regime (ξ_*s*_ < *L* < *l*_*e*_, *L*/ξ_*s*_ ∼ 2.5), where *l*_*e*_ denotes the mean-free path. Similar behavior is also observed in other devices (see Supplementary Sections S4 and S5 for details).

Based on the spatial current density *J*_*c*_(*x*), we extract the edge and bulk contributions to the supercurrent and analyze their temperature dependencies, as shown in Figure 2d. For simplicity, an average edge supercurrent is defined as *Ĩ*_*c*,edge_ = (*I*_*c*,*L*_ + *I*_*c*,*R*_)/2. Both edge and bulk states *I*_*c*_(*T*) can be fitted separately using the same Eilenberger model. For device F2_600, the fitting parameters are *D* = 0.99 (0.9997) and *R*_*N*_ = 637 Ω (535 Ω), corresponding to 20 (24) modes for the edge (bulk) components.

In a ballistic junction, the characteristic energy is determined by the smaller one between the superconducting gap Δ and the Thouless energy *E*_*Th*_. The time for charge carriers to flow through the junction is estimated to be ∼*L*/*v*_*F*_, where *v*_*F*_ is the Fermi velocity and *L* is the junction length. Thereby, the Thouless energy *E*_*Th*_ ∼ *ℏv*_*F*_/*L*.^48,49^ For a short-ballistic junction, the maximal critical supercurrent for one mode is given by *I*_*c*_ = *e*Δ/*ℏ*,^35^ which equals 148 nA for *T*_*c*_ = 4 K. From the Eilenberger fitting, we estimate a critical current $I_{c}^{0}$ of 52 nA for a single channel in the 600 nm junction. Thus, the average number of channels per edge can be estimated to be 1 μ*A*/52 nA ∼20 modes. A similar estimation can be done for the 800 and 1000 nm junctions on flake F2, where the number of channels per edge is 6 and 8 modes, respectively (see Supplementary Sections S4 and S5). Note that the number of modes is estimated by considering all channels to be normal spin-degenerate 2π modes. If these are 4π modes in an intermediate long junction, the number of modes should be reduced by a factor between 1 and 2. When a junction is in the long-junction regime, the product *I*_*c*_ *R*_*N*_ ∝ 1/*L*. In Figure 2e, we plot the *I*_*c*_ *R*_*N*_ products of all three junctions on F2, which match well to the linear trend. To demonstrate the generally high transparency of the junctions, we plot the critical currents *I*_*c*_ of all three junctions on the same BiSb flake as a function of junction length *L* in Figure 2f, alongside simulated curves based on the Eilenberger theory. All data points lie between the theoretical curves corresponding to interface transparencies *D* = 0.9 and *D* = 1, indicating that *I*_*c*_ is dominated by the consistently high transparency modes across all devices.

### 2.3 Topological Protection: 4π-Periodic Supercurrent Correlation

To verify the non-trivial topological nature of the hinge modes, we performed radio frequency (RF) measurements to investigate the Shapiro steps. In conventional Josephson junctions with a 2π-periodic current–phase relation (CPR), the voltage steps are quantized as *V* = *nhf*/2*e*, where all integer steps appear. In contrast, in the presence of topologically protected gapless Majorana zero modes (MZMs), the CPR becomes 4π-periodic, resulting in the suppression of odd Shapiro steps, a hallmark of the fractional a.c. Josephson effect.^14^ The observation of missing odd steps, particularly beyond the first one, has been reported as a characteristic signature of a topological 4π-periodic supercurrent.^19,45,50,51^ Nevertheless, it is also recognized that a 4π-periodic CPR may arise in non-topological scenarios. For example, specific parameter regimes of the resistively, capacitively and inductively shunted junction (RCLSJ) model can mimic such behavior,^52^ and extremely high junction transparency can also lead to similar effects in trivial systems.^53^ Therefore, it is crucial to demonstrate a direct correlation between the observed 4π-periodic signals and the underlying topological states in a wide-range parameter space.

In Figure 3a, we show the Shapiro step binning map for device F2_600 under 0.9 GHz irradiation, where the first and third steps (*n* = 1 and 3) are absent, as indicated by white arrows. The missing-step phenomenon is measurable from 0.6 GHz, and these steps reappear at higher microwave frequencies (Supplementary Section S6). The suppression of the odd steps can be phenomenologically quantified by the *Q*_*i*,*i*+1_ factor, where *i* is an odd number. *Q*_*i*,*i*+1_ represents, in general, the ratio between the width of an odd step and that of its successive even step. When *Q*_*i*,*i*+1_ < 1, step *i* is considered as reduced. In Figure 3b, factor *Q*_12_ = *w*1/*w*2 is plotted as a function of the frequency (see Supplementary Section S13 for estimation details). The observed frequency dependence rules out alternative explanations such as Landau–Zener transitions.^51^ Moreover, at 0.9 GHz, the odd-step suppression remains visible up to 1.2 K (Supplementary Section S7), underscoring the thermal stability of the 4π-periodic component.

**Figure 3:**
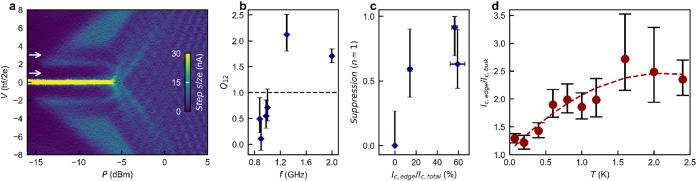
Fractional a.c. Josephson effect and topological protection of the 1D hinge modes. **a**, Shapiro steps in device F2_600 measured under radio frequency (RF) excitation of 0.9 GHz at 70 mK. The colormap shows the Shapiro step size calculated from the d.c. voltage bins as a function of d.c. voltage normalized to $\frac{hf}{2e}$ and RF power. White arrows mark the missing odd (*n* = 1, 3) Shapiro steps. **b**, Frequency dependence of the ratio between *n* = 1 and *n* = 2 step size (*Q*_12_). **c**, Suppression factor $\left(1-Q_{12}/Q_{12}^{max}\right)$ as a function of the edge-mediated supercurrent for different devices, highlighting an important observation that the *n* = 1 Shapiro step is more diminished in junctions with higher edge state contribution. This suggests overall topological protection of the 1D hinge modes. **d**, *I*_*c, edge*_/*I*_*c, bulk*_ ratio for device F2_600 showing an increasing trend with temperature up to 2 K, suggesting that the bulk *I_c_* decays more rapidly than that of the ballistic edge states. The dashed curve is guide-to-the-eye.

Fractional Shapiro steps are observed in multiple devices (Supplementary Sections S8 and S9), spanning a broad range of RCSJ model parameters (*R* ∈ [20, 30] Ω, *I*_*c*_ ∈ [0.3, 3.8] μA). This indicates that the missing steps cannot be simply attributed to a specific choice of RCSJ parameters. The reappearance of the odd Shapiro steps is assumed to occur when the RF excitation energy becomes comparable to the characteristic energy scale associated with the 4π-periodic supercurrent, i.e., $\frac{hf}{2e}\approx I_{c}^{4\pi }R_{N}$ . From the *Q*_12_ versus frequency dependence in Figure 3b, we extract a crossover frequency of *f*_*c*_ ∼ 1.5 GHz. Using this value, the magnitude of the 4π-periodic supercurrent can be estimated as $I_{c}^{4\pi }=\frac{hf_{c}}{2eR_{N}}\approx 150\;\mathrm{nA}$ , where *R*_*N*_ = 20 Ω is the experimentally measured normal-state resistance. We observe missing odd steps down to *f*_min_ ≈ 0.6 GHz (Supplementary Section S6), which implies a lower bound on the parity lifetime of 4π-periodic Andreev bound states, τ_*p*_ ≳ 1/*f*_min_ ≈ 1.6 ns.

As an important step, we test the correlation between the presence of the edge state and the visibility of the fractional Shapiro steps. We define a normalized suppression factor $\gamma =1-Q_{12}/Q_{12}^{max}$ for all junctions. $Q_{12}^{max}$ is the maximal value of *Q*_12_ among all measured junctions and frequencies. If γ = 1, it means that the first step is fully suppressed; oppositely, if γ = 0, there is no suppression of the odd steps. In Figure 3c, we plot the suppression factor γ as a function of the estimated percentage of the edge supercurrent in various junctions. A positively correlated behavior can be found clearly, suggesting that the hinge states are the topological origin of the observed fractional Shapiro steps.

As we demonstrated before, both the bulk and edge channels are ballistic. However, the hinge modes, due to their topological protection, are expected to exhibit enhanced robustness against thermal noise. We assess this robustness by examining the temperature dependence of the ratio between edge and bulk critical currents, defined as α = *I*_*c*, edge_/*I*_*c*, bulk_, as shown in Figure 3d, where *I*_*c*,edge_ = (*I*_*c*,*L*_ + *I*_*c*,*R*_). For device F2_600, α displays an increasing trend up to 2 K, suggesting that the edge modes maintain their coherent transport while thermal effects increasingly suppress the bulk contribution. We attribute the robustness of the topological states against temperature to spin–momentum locking, which suppresses backscattering in the normal state and consequently enhances transparency and the mean-free path.^54^ Unlike ref 54, the induced gap difference between the topological edge and bulk states cannot be resolved directly from the temperature dependence *I*_*c*_(*T*), likely due to the similar ballistic nature of transport in both channels. Nevertheless, the extracted edge-to-bulk contribution (α) ratio indicates a trend consistent with a larger induced superconducting gap in the edge states, which may contribute to the enhanced temperature robustness of the topological edge channels. Similar trends in α were consistently observed across other devices (Supplementary Sections S4 and S5), providing compelling evidence for the existence of topologically protected 1D hinge modes in Bi_0.97_Sb_0.03_.

### 2.4 Multiple Hinge Modes Pinned to Structural Irregularities

We now turn our attention to the multi-edge channels. As discussed previously, the observed supercurrent must be carried by multiple (broadened) edge channels, which appears to deviate from the SOTI prediction of a single hinge mode per edge. A plausible explanation lies in the structural imperfections of exfoliated flakes: side edges are rarely atomically flat or perfectly aligned with a single crystallographic facet. Instead, steps, discontinuities, and other irregularities often form along the edge, effectively introducing additional internal boundaries that can host localized hinge modes.^55,56^

We directly observed this effect in a junction with an additional step. In Figure 4a, a scanning electron microscopy (SEM) image shows the side-view junction F2_1000. A 15 nm step was found on the top surface. To reproduce the measured *I*_c_(*B*) of this junction, it is necessary to include both the step feature and an additional hinge current density at the step in the model, resulting in a strong asymmetric current distribution (Figure 4b, shaded area). This is qualitatively in good agreement with the extracted supercurrent profile (Figure 4b, red dotted line).

**Figure 4:**
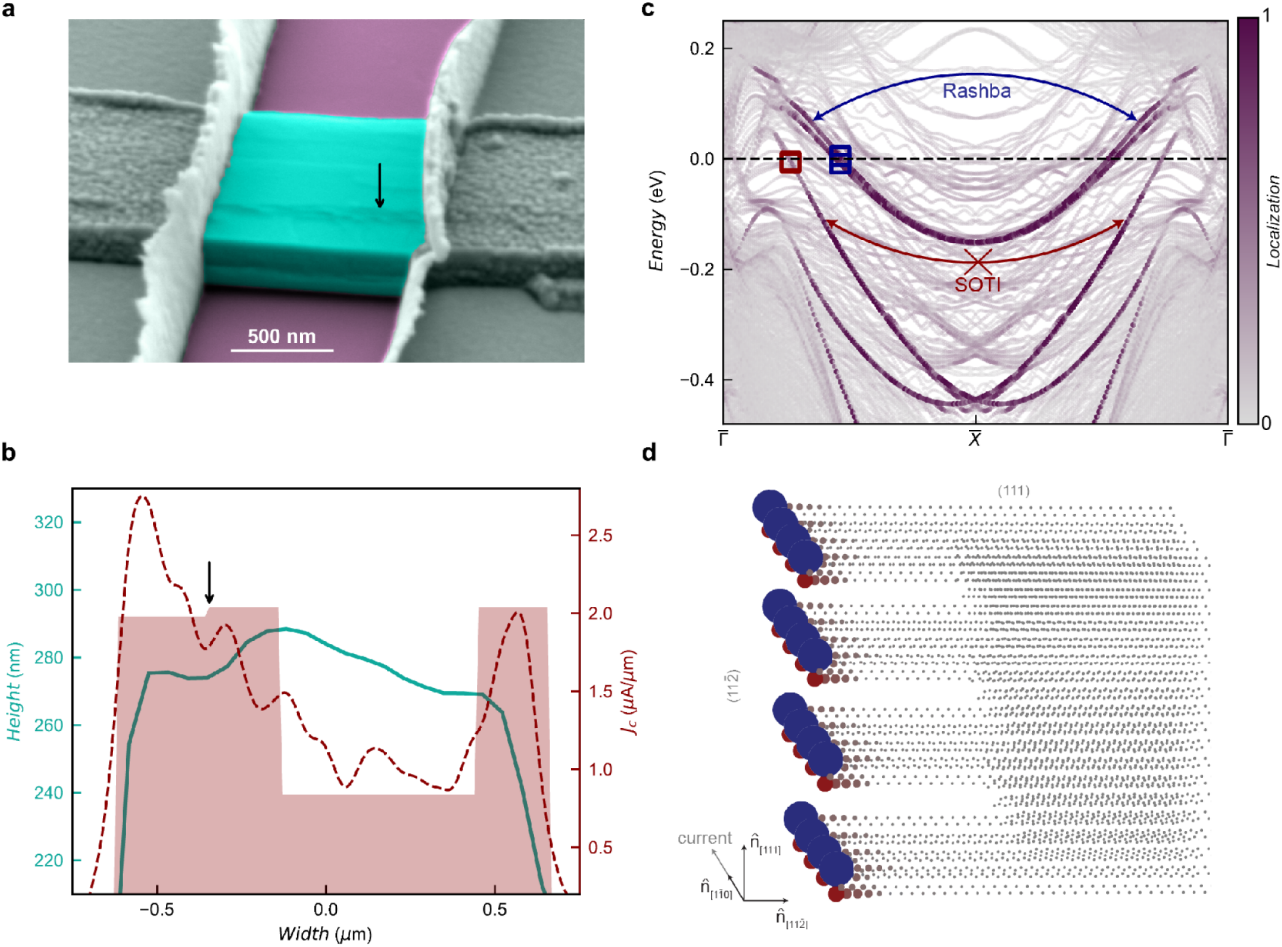
Experimental and theoretical signatures of the effect of multiple structural irregularities on hinges. **a**, Cross-sectional SEM image of the F2_1000 device showing the additional step (black arrow) present near one of the junction edge (also visible in AFM image, Supplementary Section S11). **b**, Overlayed AFM height profile (solid turquoise) showing the ∼15 nm high step near the left edge of the junction, designed $J_{c}^{\mathrm{set}}\left(x\right)$ model (shaded region) including this step (black arrow), and experimental $J_{c}^{\exp}\left(x\right)$ distribution (dashed dark red) showing the enhanced width of the left edge due to this step. $J_{c}^{\mathrm{set}}\left(x\right)$ is scaled as per the $J_{c}^{\exp}\left(x\right)$ data. **c**, Tight-binding band structure of a 20 bilayer Bi_0.97_Sb_0.03_ slab (8 nm thick by 16 nm wide) with translation symmetry in the [11̅0] direction, confinement along the top (111) and side (112̅) surfaces, and with four identical artificial step edges on one side. The color intensity represents the localization defined as the sum of the wave function density over the 10 sites around the site where the wave function density is maximal. The most strongly localized bands are four times degenerate (Rashba). **d**, The summed wave function density of the highlighted degenerate Rashba (blue) and SOTI (red) modes at the Fermi level (*E*_F_ = 0).

To investigate this phenomenon, we perform tight-binding (TB) calculation using Kwant^57^ on the Bi_0.97_Sb_0.03_ crystal, based on the 16-band tight-binding model of Liu and Allen.^58^ We construct a slab geometry with translational symmetry along the [11̅0] direction and confinement along the top (111) and side (112̅) surfaces. Structural irregularities are introduced by forming four identical step edges along the (112̅) surfaces. The resulting band structure is shown in Figure 4c, where the highlighted bands are strongly localized at the hinges and their wave function density at the Fermi level is plotted in Figure 4d. This confirms our idea that the steps can activate new hinge modes.

However, this alone cannot fully account for the generally broadened edge current profile (approximately 100–200 nm). From the simulations, one can see that the activated SOTI states remain well localized within only a few nanometers from the hinge. A closer inspection of the band structure in Figure 4c reveals that the set of localized hinge states contains (i) a pair of opposite-spin Rashba modes, both connecting only conduction bands and (ii) a single-spin SOTI mode, connecting conduction and valence bands. Crucially, we classify the SOTI mode as localized, helical modes that connect valence and bulk bands. Both states are 4-fold degenerate due to the identical step structure, which is chosen to keep the dispersion clean and to directly relate the observed degeneracy to the number of steps.

The coexistence of both Rashba and SOTI 1D states is only visible when a real crystal lattice is taken into account. This leads to a clearer interpretation of the commonly observed broadened edge current: Both states can carry supercurrent. A Rashba mode at momentum *k* can backscatter to the state at −k + δk which has the same spin and is localized on the same hinge, directly leading to the delocalization of the states and therefore a broadening of the edge current. Such Rashba-type states localized at the edge have been theoretically proposed^59,60^ and experimentally observed in STM and ARPES measurements on Bi-based systems.^61–63^ However, distinguishing between the helical and Rashba states has remained challenging in these types of experiments. Although both types of states can contribute to the edge supercurrent, the SOTI states are topologically protected, whereas the Rashba states are not. Consequently, only the SOTI states are relevant to the observed 4π component. From the frequency dependence, the 4π supercurrent fraction is estimated to be about 8%, consistent with this picture, corresponding to only 2–4 SOTI channels among approximately 35–40 Rashba-type channels.

Another peculiar feature that we have observed is the quasi-1D transport in bulk states. To obtain a better understanding of the size effect, we carried out measurements on junctions with different sizes. A striking width dependence was found. In Figure 5a−c, we plot the *I*_c_(*B*) of three different junctions that cross the entire flake (namely, the edge JJ as defined previously) with widths W = 1800, 700, and 300 nm, respectively. As the width decreases, the measured SQI pattern changes from a clear SQUID-like shape to a monotonic decay. This confirms our idea that in these long junctions, besides the hinge modes, the bulk supercurrent is carried by the bands with an extremely small wave vector *k*_F_, most likely from the L-pocket (Supporting Information Section 16), which are quantized even in the submicrometer-sized flakes.

**Figure 5:**
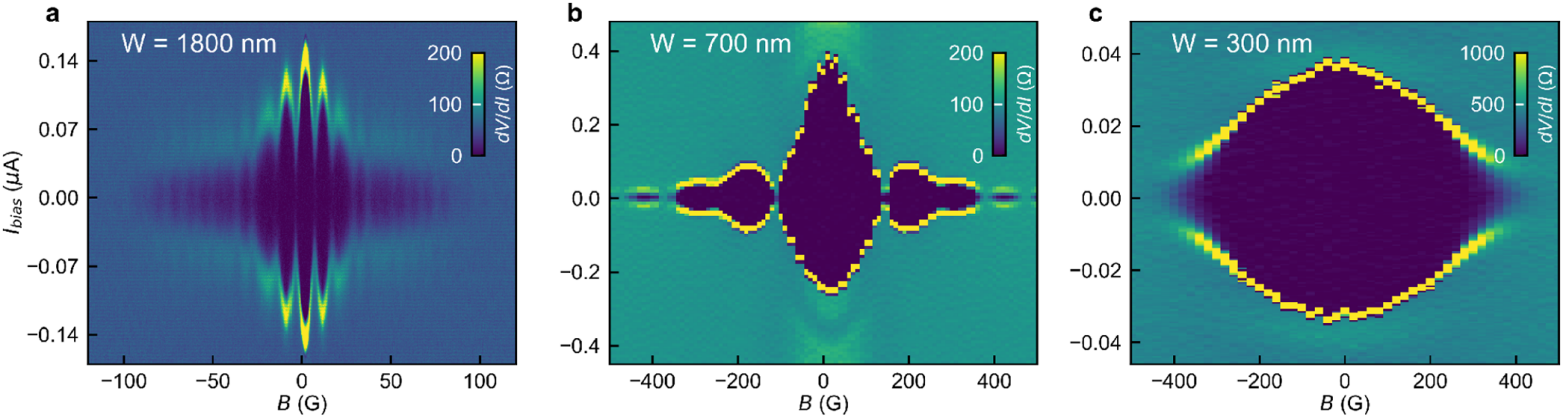
Finite-size effect on the SQI pattern. **a−c**, Comparing the *I_c_* (*B*) for **a**, *W* = 1800 nm (300 mK), **b**, *W* = 700 nm (100 mK), and **c**, *W* = 300 nm (100 mK) wide junctions reveals the quantum confinement effect due to the width of an edge JJ. A gradual shift from the SQUID-like response in the widest flake to a monotonically decaying *I_c_* (*B*) in the narrowest flake was observed.

More importantly, the edge current disappears in the 300 nm-wide junction. This observation is consistent with the estimated edge width, suggesting that the extended edge states on the two sides can couple to each other, leading to the opening of an effective gap in the Rashba state. Although previous theoretical studies have suggested that the finite-size intercoupling between opposite topological hinge modes can open a gap in the one-dimensional helical states,^12^ we argue that the calculated wave functions of all hinge states are strongly localized within only a few nanometers. Therefore, such coupling based on the direct overlap of hinge-state wave functions is unlikely. We speculate that the apparent disappearance of the hinge-related supercurrent is instead mainly a consequence of the reduced transparency to the topological hinge states when most of the surrounding Rashba states become gapped. However, such a claim can only be fully supported through dedicated theoretical analysis and additional experiments aimed at clarifying the role of trivial Rashba states in facilitating the experimental visibility of non-trivial SOTI states in SQI measurements, which is out of scope for the present study.

Our findings highlight that a key challenge in SOTIs such as Bi_0.97_Sb_0.03_ is the hybridization between topological hinge states and bulk or trivial Rashba states, which reduces spectral isolation but does not suppress the robust 4π-periodic Josephson signal. Possible strategies to mitigate coexisting bulk channels include local electrostatic gating near the hinges, reducing bulk contributions via thinner flakes, selective superconducting coupling to hinge modes, and tuning via magnetic anisotropy or strain. These approaches may enable improved isolation and control of 1D SOTI states, supporting their potential for Majorana-based quantum devices.

## 3 Conclusion

We have observed robust hinge states in Bi_0.97_Sb_0.03_ single crystals, with results that are highly reproducible. Importantly, our work demonstrates, for the first time, a direct correlation between the 4π-periodic Josephson effect and the presence of hinge modes, thereby confirming their topological nature. Furthermore, both theoretical and experimental results show that multiple hinge states can be activated by the step-like structure of the crystal. This firmly establishes the Bi_1–*x*_Sb_*x*_ alloy system as a promising platform for advancing topological quantum devices, opening new opportunities for quantum network-based architectures through nanoengineering of artificial one-dimensional edges. The emergent one-dimensional Rashba states are identified as the primary origin of the extended edge current distribution. This not only provides a comprehensive explanation for the widely observed broadened edge profiles across various SOTI systems, but also opens new possibilities for designing and implementing topological devices that leverage Rashba-state–dominated edge transport.

## 4 Methods

### 4.1 Crystal Growth

Bi_1–*x*_Sb_*x*_ single crystals are grown using a modified Bridgman method. High-purity Bi ingots (99.999%) and Sb ingots (99.9999%) were packed in a cone-shaped quartz tube and sealed under vacuum (4 × 10^–7^ mbar). The tube was first put in a box furnace and heated up to 600 °C for 12 h. The tube was shaken several times to obtain a homogeneous mixture of Bi and Sb. Then, the tube was quickly cooled to room temperature and hung vertically in a mirror furnace for crystal growth. The tube was heated to 300–400 °C, starting from the cone-shaped bottom, and the molten zone was translated up at a rate of 1 mm/hour. Flat crystals up to 1 cm in length were obtained by cleaving the crystal boule.

### 4.2 Device Fabrication

We performed micromechanical exfoliation of Bi_0.97_Sb_0.03_ single crystals to transfer high-quality flakes on pre-cleaned Si/SiO_2_ substrates with a 120 nm oxide thickness. The thickness of the flakes was measured by using a commercial atomic force microscope (Icon, Bruker). Josephson junctions were fabricated on the desired flakes using standard electron-beam lithography, followed by Ar^+^ etching at 300 W RF power for 30 s to remove native oxide and contamination layers before in situ sputter deposition of 120 nm Nb electrodes and 2 nm of Pd as a capping layer to protect the Nb from oxidation. Nb d.c. sputtering was performed at a relatively slow rate, 10 nm/min, to mitigate the Ar-plasma-induced degradation of the BiSb flakes.

## Supplementary Material



## Data Availability

All data needed to evaluate the conclusions in the paper are present in the paper and the Supporting Information. The experimental data sets that can be used to reproduce the findings of this study are available via Zenodo (10.5281/zenodo.21457097). A preprint of this manuscript has been submitted to arXiv.^64^

## Notes

Superconducting quantum interference model; tight-binding model simulation; step-by-step scheme to estimate I_c_, edge and I_c_, bulk; supercurrent characteristics of F2_800 nm Josephson junction; supercurrent characteristics of F2_1000 nm Josephson junction; RF dependence of missing odd Shapiro steps in F2_600 nm junction; temperature dependence of missing odd Shapiro steps in the F2_600 nm junction; missing odd Shapiro steps in the F2_800 nm junction; supercurrent characteristics of the F1 bulk-edge Josephson junction device; monotonically decaying I_c_B for different bulk Josephson junctions; surface morphology of the flake F2 probed with atomic force microscopy; SEM characterization; Q_12_ calculation in RF Shapiro steps measurements; supercurrent characteristics of a shorter (L = 350 nm) Josephson junction; Table: summary of device parameters and properties; Brillouin zone schematic of Bi_0.97_Sb_0.03_ in momentum space; energy-dispersive X-ray spectroscopy (EDS) of the Bi_0.97_Sb_0.03_ crystal (PDF)
