# Supplementary material for: Coexisting Topological Hinges and 1D Rashba States in Bi0.97Sb0.03 Revealed by the Josephson Effect
Source: ACS Nano. 2026 Jul 29;20(31):21636–45. doi: 10.1021/acsnano.6c01686 (PMC13472327; doi:10.1021/acsnano.6c01686)
Supplement: Supplementary file 1 [file nn6c01686_si_001.pdf]

Coexisting topological hinges and 1D Rashba states in $Bi_{0.97}Sb_{0.03}$ revealed by the Josephson effect

Biplab Bhattacharyya[1], Stijn R. de Wit[1], Zhen Wu[1], Yingkai Huang[2], Mark S. Golden[2], Alexander Brinkman[1], Chuan Li[1*]
[1]MESA+ Institute, University of Twente, Enschede, the Netherlands
[2]Van der Waals–Zeeman Institute, IoP, University of Amsterdam, Amsterdam, the Netherlands.

Supplementary Information

Contents

1 Superconducting quantum interference model 2

2 Tight-Binding Model Simulation 7

3 Step-by-step scheme to estimate $I_{c,\ edge}$ and $I_{c,\ bulk}$ 9

4 Supercurrent characteristics of F2_800 nm Josephson junction 11

5 Supercurrent characteristics of F2_1000 nm Josephson junction 12

6 RF dependence of missing odd Shapiro steps in F2_600 nm junction 13

7 Temperature dependence of missing odd Shapiro steps in F2_600 nm junction 14

8 Missing odd Shapiro steps in F2_800 nm junction 15

9 Supercurrent characteristics of F1 bulk-edge Josephson junction device 16

10 Monotonically decaying I_cB for different bulk Josephson junctions 17

11 Surface morphology of the flake F2 probed with atomic force microscopy 18

12 SEM characterization 19

13 Q_{12} calculation in RF Shapiro steps measurements 20

14 Supercurrent characteristics of a shorter ($L = 350$ nm) Josephson junction 21

15 Table: Summary of device parameters and properties 22

16 Brillouin zone schematic of $Bi_{0.97}Sb_{0.03}$ in the momentum space 23

17 Energy-dispersive X-ray spectroscopy (EDS) of the $Bi_{0.97}Sb_{0.03}$ crystal 24

1 Superconducting quantum interference model

1.1 Simulation of the Field Dependence in SNS Junctions

The field dependence of the SNS junction is simulated using a phenomenological model based on Ref.[1] In this model, we introduce an effective decay length, L_c. Instead of a $1/L$ dependence, we consider an exponential decay $J_c = J_0 \exp(-L/L_c)$. When applying the Landau gauge, a phase is acquired as the carrier moves from x_1 to x_2, given by $\frac{2\pi B_z(y_2+y_1)}{\phi_0}$.

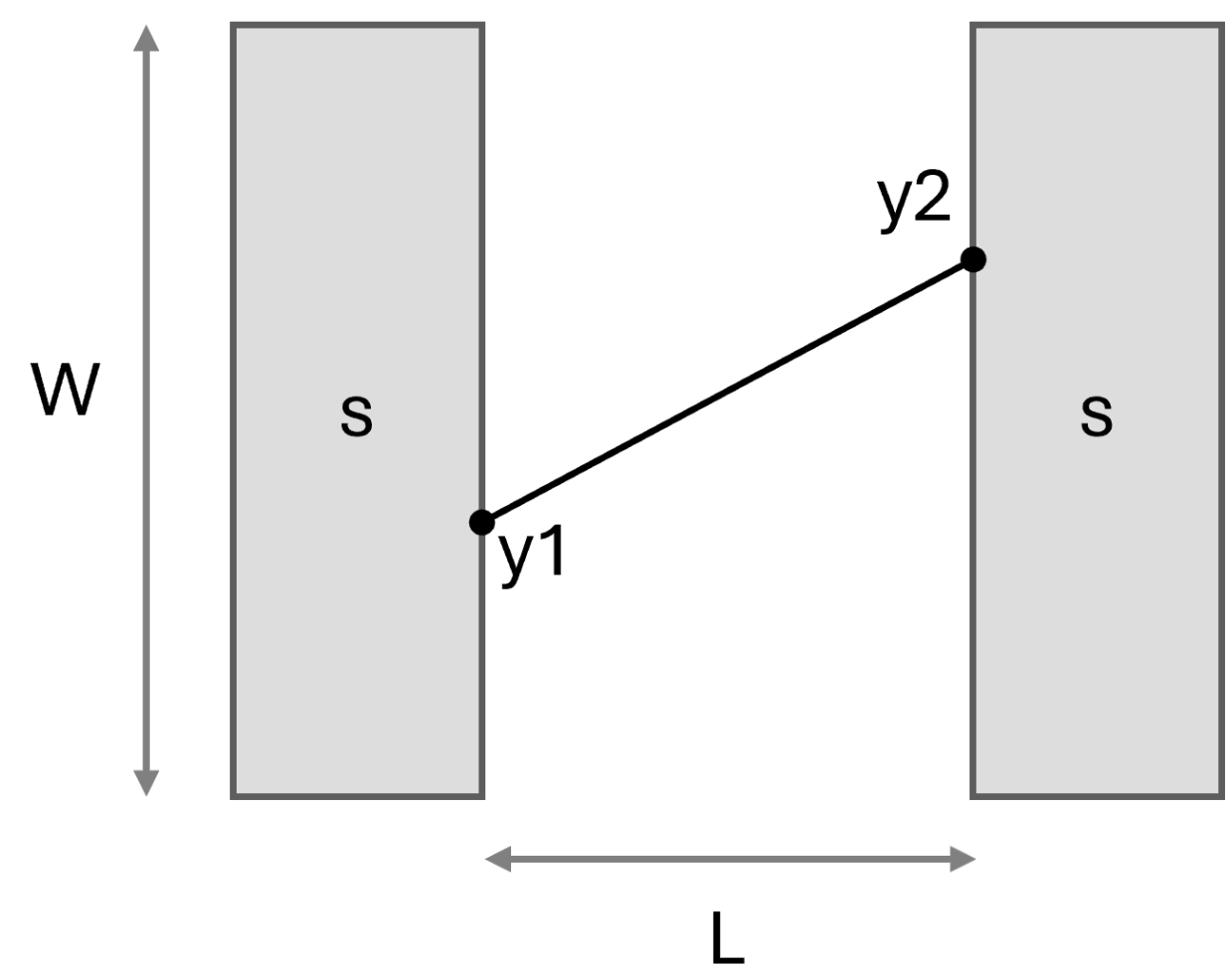

Fig S1 | Schematic of the superconducting quantum interference model.

The complete expression for the supercurrent is:

$$I(\Delta\phi_0, B_z) = \int_0^W \int_0^W \frac{1}{L^2 + (y_2 - y_1)^2} \sin(\Delta\phi(B_z))\, dy_1 dy_2, \tag{1}$$

where the total phase gained by a particle traveling from $(0, y_1)$ to (L, y_2) is given by:

$$\Delta\phi(B_z) = \Delta\phi_0 + \frac{2\pi B_z L(y_1 + y_2)}{\phi_0}. \tag{2}$$

Here, L is the effective junction length, accounting for the magnetic field focusing effect, and $\Delta\phi$ is the superconducting phase difference between the two leads. The Josephson current is obtained by varying $\Delta\phi$ and maximizing Eq. (1).

While refining the model, we identify three key findings:

1. The resolution of $J_c(x)$ depends on the decay length L_c.
2. The shape of the edge current can be smoothed, and in some cases, its width deviates from the actual distribution.
3. The model captures various details, including steps and edge currents.

1.2 Procedure for Obtaining the Optimal Edge Width

In this study, we resolve the edge states by comparing both $I_c(B)$ and the converted $J_c(x)$, obtained using the Dynes and Fulton method (explained in the next section), with simulation results. The analysis follows these steps: 1) Optimize the $I_c(B)$ simulation by minimizing the standard deviation (std) between the simulated and experimental data. 2) Convert both the simulated and experimental data into $J_c(x)$, then compute the std and uncertainties. 3) Extract the edge current percentage from the converted $J_c(x)$ by varying the edge width and compare it to the original edge current percentage defined in the model.

1.2.1 Optimization of $I_c(B)$ for Simulation

Based on the measured $I_c(B)$, we compute $J_c^{\text{exp}}(x)$. A simple $J_c(x)$ model, consisting of both edge and bulk currents, is then designed. By tuning parameters such as edge width and bulk current density, we optimize the results by minimizing the std.

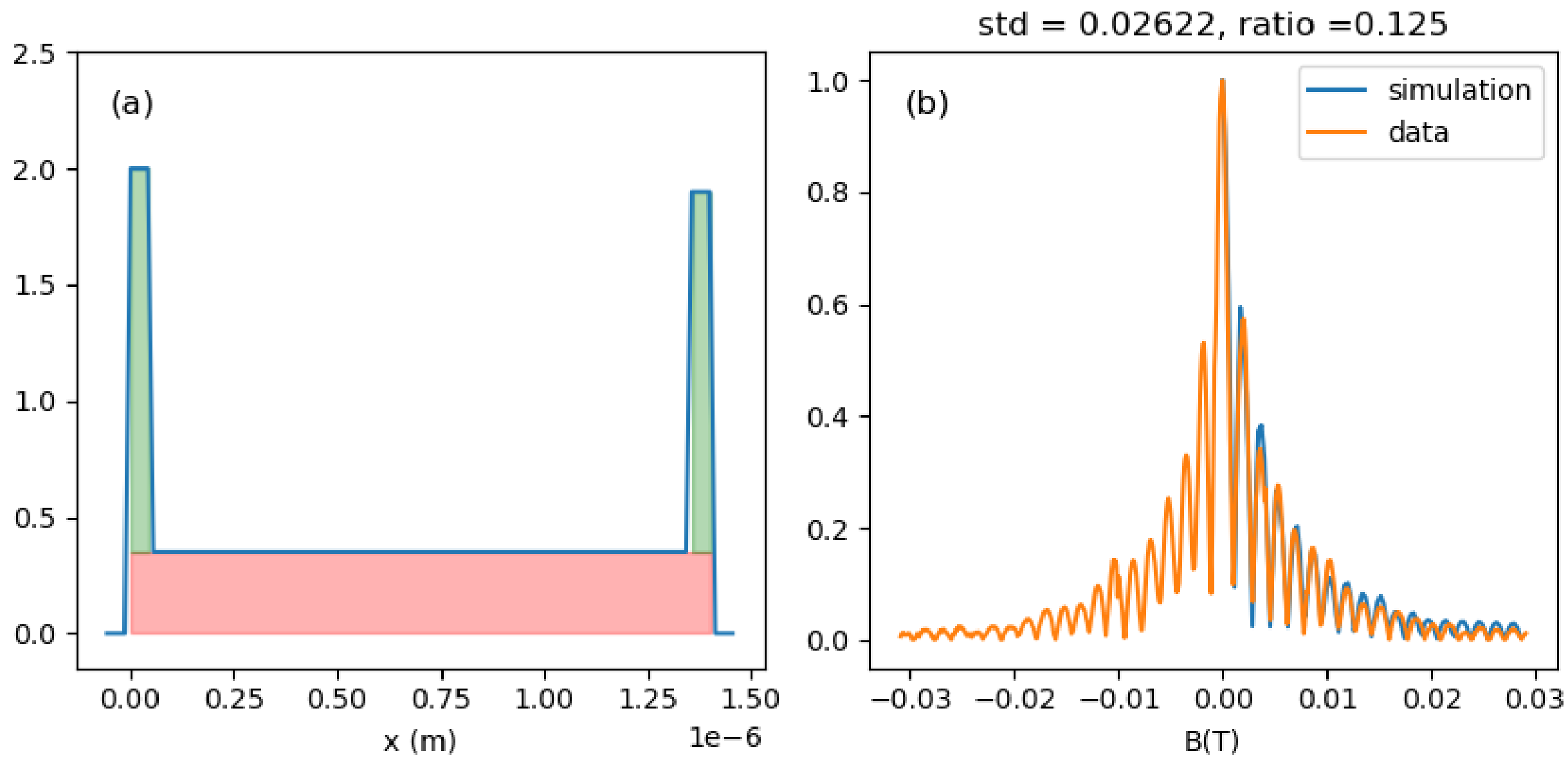

Fig S2 | Optimization of the simulation for a 600 nm junction. (a) Designed current distribution: red represents bulk current, and green represents edge current. (b) Comparison between simulated and experimental $I_c(B)$ data. The standard deviation is displayed on top.

1.2.2 Computation of $J_c(x)$ and Uncertainty Estimation

Once $I_c(B)$ is obtained, the current distribution $J_c(x)$ is calculated for both the simulation and experimental data. The std is then computed, and the uncertainty in current is determined by: $\sigma = \text{std} \times I_{\text{tot}}$.

1.2.3 Estimation of the Edge Width from the Simulation

A notable feature of the edge current is that the calculated current distribution appears smoothed. For instance, the computed $J_c^{\text{sim}}(x)$ for the sharp edge current distribution shown in Fig. S2(a) is presented in Fig. S3(a).

The actual edge width is no longer easily discernible by eye. To address this, we compare the percentage of the edge current in the calculated $J_c(x)$ at different widths ($P_{\text{edge,sim}}$) with the designed edge current percentage ($P_{\text{edge,set}}$) (Fig.S2(a)). The procedure for determining $J_c(x)$ is

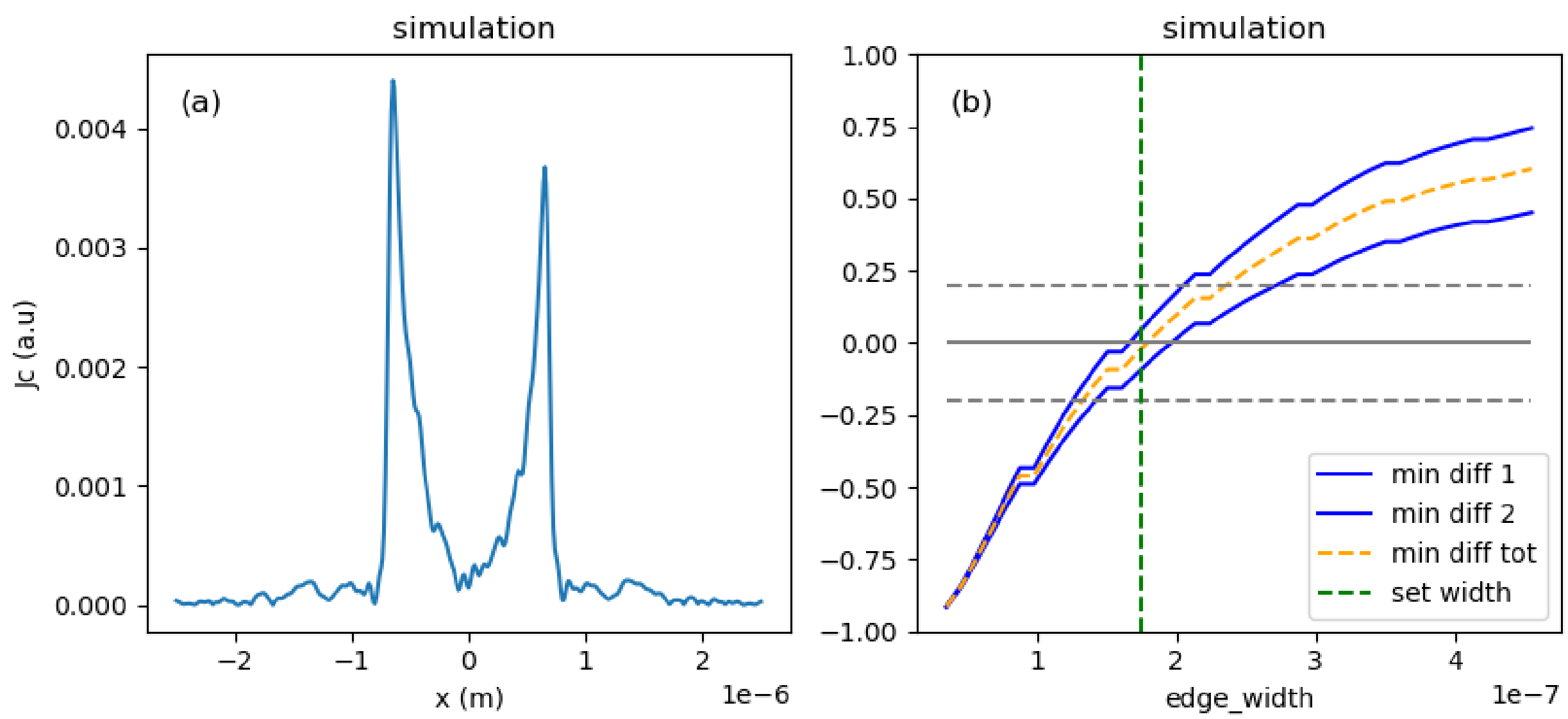

Fig S3 | Estimation of the edge width.

outlined in Section 3. Ultimately, we plot the relative difference $(P_{\text{edge,sim}} - P_{\text{edge,set}})/P_{\text{edge,set}}$, as shown in Fig. S2(b). From this analysis, we identify an optimal edge width for a given set of parameters at approximately 250 nm, where the dashed orange line crosses zero. Notably, this optimal width is larger than the originally designed edge width (green dashed line). This estimation method is applied to all presented devices.

1.3 Averaging Effect in Resolving Current Distribution

During the simulation, we observed several effects related to the model that may be useful for broader applications, including its limitations.

The effect of the decay length L_c is illustrated in Fig. S4. All three simulations use the same geometry and designed current distribution (a simple homogeneous distribution), differing only in the choice of L_c. For relatively large L_c, a blurring effect occurs, causing a smoothing of the calculated $J_c(x)$. As L_c decreases, the sharpness of the reconstructed $J_c(x)$ significantly improves.

This effect is even more pronounced for edge currents. As shown in Fig. S5, when L_c is

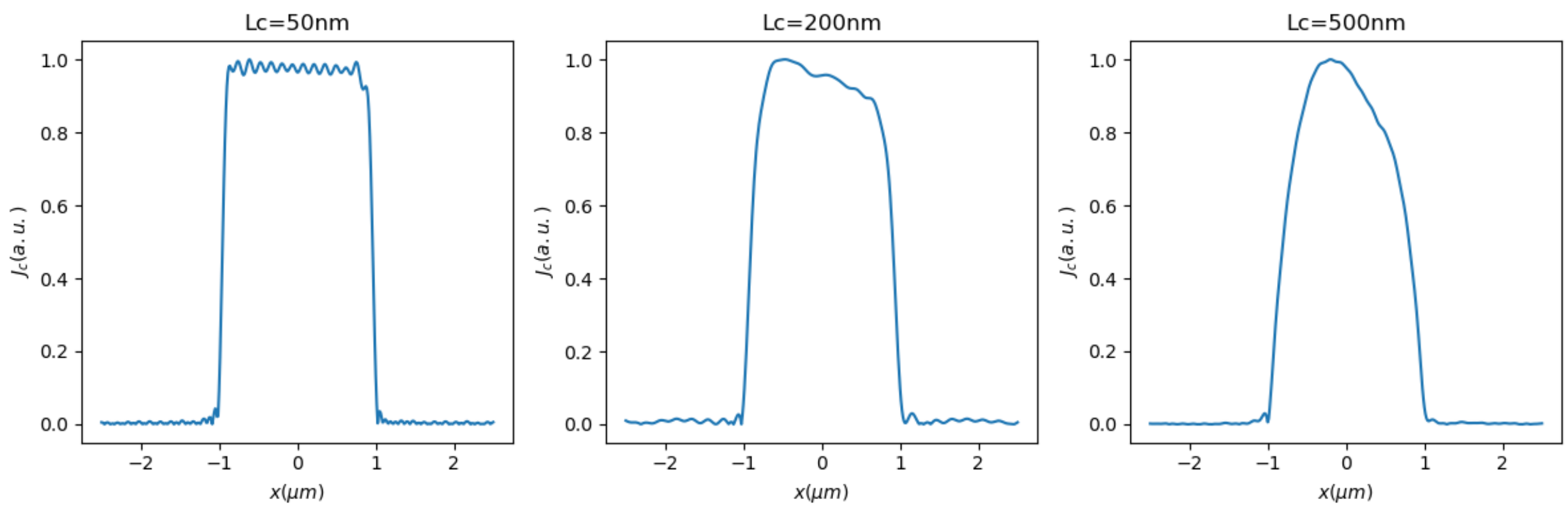

Fig S4 | Estimation of the edge width.

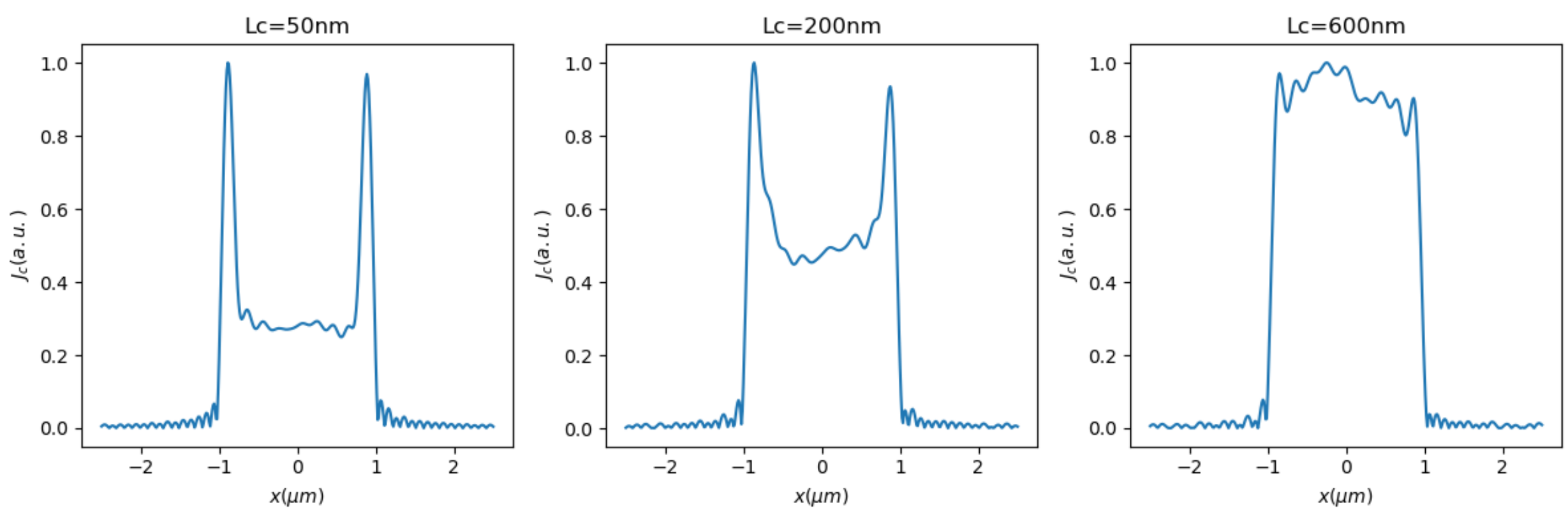

Fig S5 | Impact of decay length on edge current resolution.

relatively large, the edge current can be almost entirely suppressed (panel a). Therefore, accurately estimating the edge current (as described in the previous section) is essential before extracting it from experimental data.

Intuitively, the decay length L_c acts similarly to a coherence length, facilitating interference across the sample width and effectively averaging out the current density distribution.

2 Tight-Binding Model Simulation

To visualize the hinge states in $Bi_{1-x}Sb_x$ and compare them to experimental results, we simulate $Bi_{1-x}Sb_x$ using the 16-band tight-binding model[2] on a lattice. We first reproduce the eigenvalues at the high-symmetry points (HSPs) for Bi and Sb, as presented in Ref.,[2] by solving the continuous tight-binding model. Next, we construct the crystal lattice using Kwant[3] with the same parameters. To validate our approach, we successfully reproduce the band structure in the $[1\bar{1}0]$ direction of a bilayer bismuth system, as reported by Shuichi Murakami,[4] as shown in Fig. S6a.

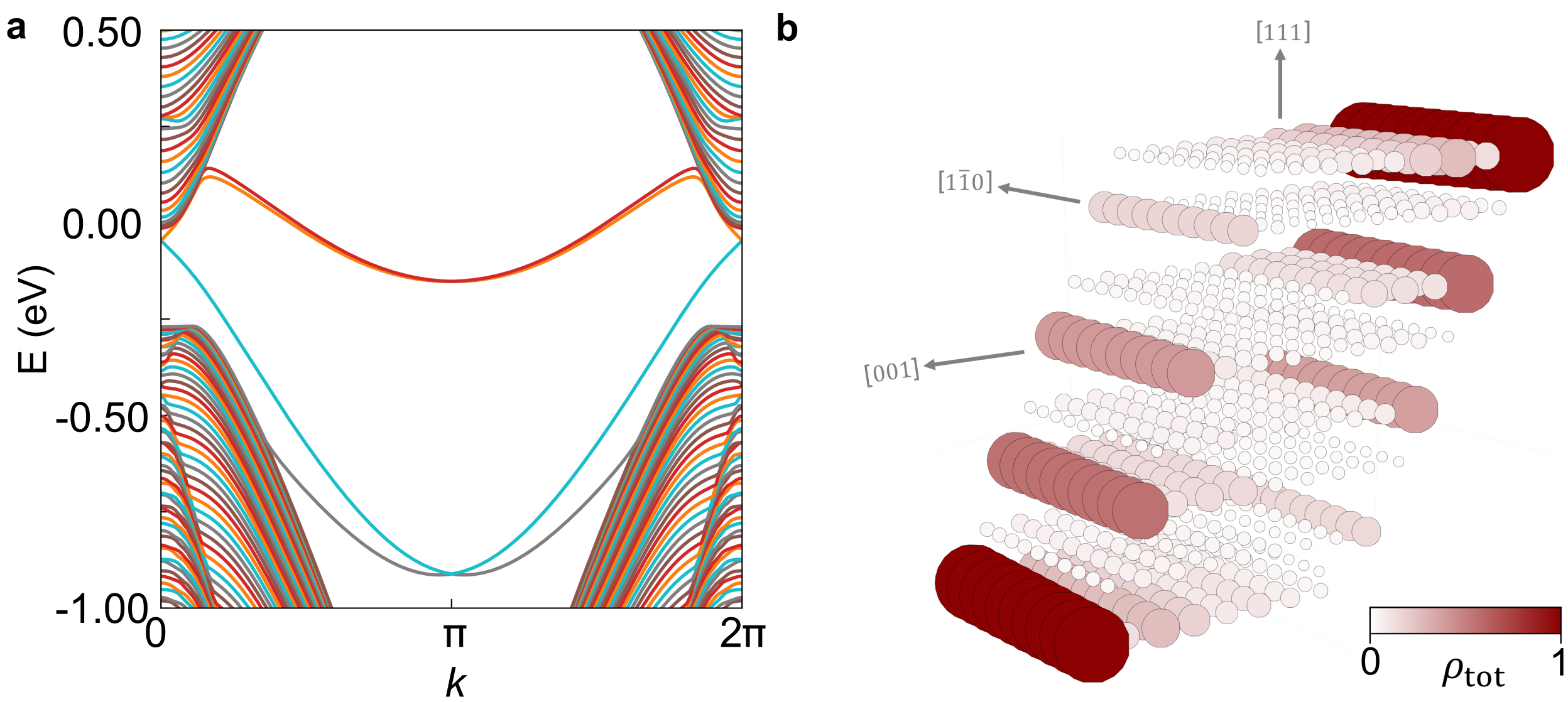

Fig S6 | **a**, Band structure of a bismuth bilayer with translational symmetry in the $[1\bar{1}0]$ direction. **b**, visualization of the hinge modes in the $[1\bar{1}0]$ direction in a 3D slab confined by top and side surfaces with their normals in the $[111]$ and $[001]$ direction respectively. Site color and size indicate the summed density of the modes at the Fermi surface, ρ_{tot}.

Using Kwant, we perform calculations for two types of models: 1) a finite-size 3D model, 2) an infinite-size model. In both cases, we confirm the existence of robust hinge modes across various facet configurations. The model parameters for $Bi_{1-x}Sb_x$ are interpolated as $a = a_{\text{Bi}}(1-x) + a_{\text{Sb}}x$, $x = 0.03$. A direct way to visualize hinge modes is by computing the wavefunctions of the topological states at the Fermi surface and plotting their spatial distribution within the crystal

lattice. In our exfoliated flakes, the natural cleaving facet is always (111), occurring between two bilayers due to the stronger intralayer bonding within a bilayer compared to interlayer bonding. We refer to this stacking configuration as AB-AB, where one layer consists of a single AB bilayer. In Fig. S6b, the hinge states are shown for an eight-bilayer lattice. The hinges are evident and decay both along the (001) plane away from the corners and into the bulk. As seen in the simulation, the summed wavefunction remains unchanged along the hinge mode direction. This behavior arises because, in the 3D model, adding a lead imposes translational symmetry along that $[1\bar{1}0]$ direction. To explore larger cross-sections while reducing computational costs, we extend the model to the infinite system discussed in the main text. In an infinite model, it suffices to analyze the cross-section perpendicular to the translational symmetry direction.

3 Step-by-step scheme to estimate $I_{c,\ edge}$ and $I_{c,\ bulk}$

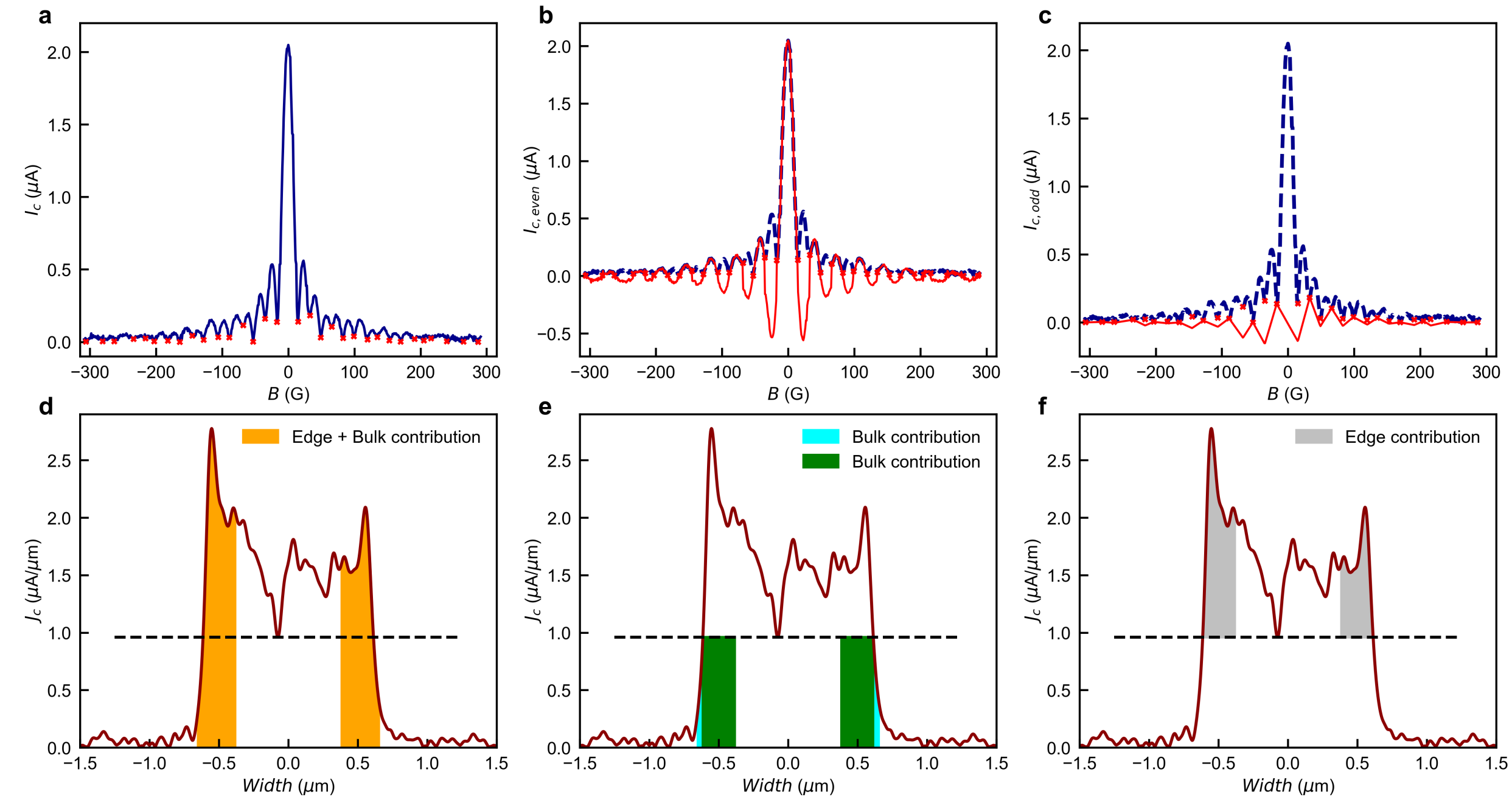

Fig S7 | **a**, An experimentally measured I_cB with red colored cross marking the position of the nodes. **b**, The even component (red solid curve) is calculated by flipping the sign of every second lobe of I_c (darkblue dashed curve). **c**, The odd component (red solid curve) is calculated by interpolating between the alternating nodes of I_c (dark blue dashed curve) but flipping sign of the intermediate node to ensure asymmetry across $B = 0$. **d**, J_c distribution as a function of width of the junction showing peaks at the edges due to presence of hinge modes. Orange shaded region highlights the area under $J_c(x)$ curve, spanning only the edge states width as estimated from the SQI model described in Supplementary Section 1. This area provides the contribution of both edge states and background bulk, $I_{c,\ (edge\ +\ bulk)}$, to the peak at junction edges. Dashed black line is the minimum value that $J_c(x)$ takes within the physical width of the junction. **e**, $J_c(x)$ profile highlighting only the bulk contribution $I_{c,\ bulk}$ to the peak at junction edges as the area under $J_c(x)$ but below the dashed black line. **f**, $J_c(x)$ profile highlighting only the edge states contribution (gray shaded region), $I_{c,\ edge} = I_{c,\ (edge\ +\ bulk)} - I_{c,\ bulk}$, to the peak at junction edges as the area under $J_c(x)$ but above the dashed black line.

As mentioned in the main text, in a JJ with out-of-plane perpendicular B, the I_cB modulation depends strongly on the $J_c(x)$ distribution in the junction expressed by the inverse Fourier transform equation:

$$I_c(B) = \left| \int_{-\infty}^{\infty} J_c(x) \exp\left(i \frac{2\pi L_{\text{eff}} B x}{\Phi_0} \right) dx \right| \tag{3}$$

where symbols have same meaning as described in main text. Dynes and Fulton[5] introduced

Fourier techniques to retrieve the $J_c(x)$ from the experimentally measured I_cB. The overall supercurrent is composed of both the even and odd parts, i.e. symmetric (cosine) and asymmetric (sine) components.

$$I_{c,\ even}(B) = \int_{-\infty}^{\infty} J_{c,\ even}(x)\ cos(\frac{2\pi L_{\text{eff}} Bx}{\Phi_0})\, dx\ ;\ I_{c,\ odd}(B) = \int_{-\infty}^{\infty} J_{c,\ odd}(x)\ sin(\frac{2\pi L_{\text{eff}} Bx}{\Phi_0})\, dx \tag{4}$$

For a symmetric I_cB distribution, the odd component of Eq. (3) is zero such that total supercurrent can be expressed with only even component $I_{c,\ even}(B)$. Whereas in presence of an odd component $I_{c,\ odd}(B)$, the total I_c can be expressed with a complex equation:

$$I_c(B) = I_{c,\ even}(B) + iI_{c,\ odd}(B) \tag{5}$$

The Fourier transform of the complex $I_c(B)$ from Eq. (5) provides the $J_c(x)$ distribution in the junction expressed as:

$$J_c(x) = \alpha \left| \int_{-B}^{B} I_c(B) \exp\left(-i\frac{2\pi L_{\text{eff}} Bx}{\Phi_0}\right)\, dB \right| \tag{6}$$

where α is the scaling factor calculated using the experimentally measured maximum I_c value at $B = 0$.

4 Supercurrent characteristics of F2_800 nm Josephson junction

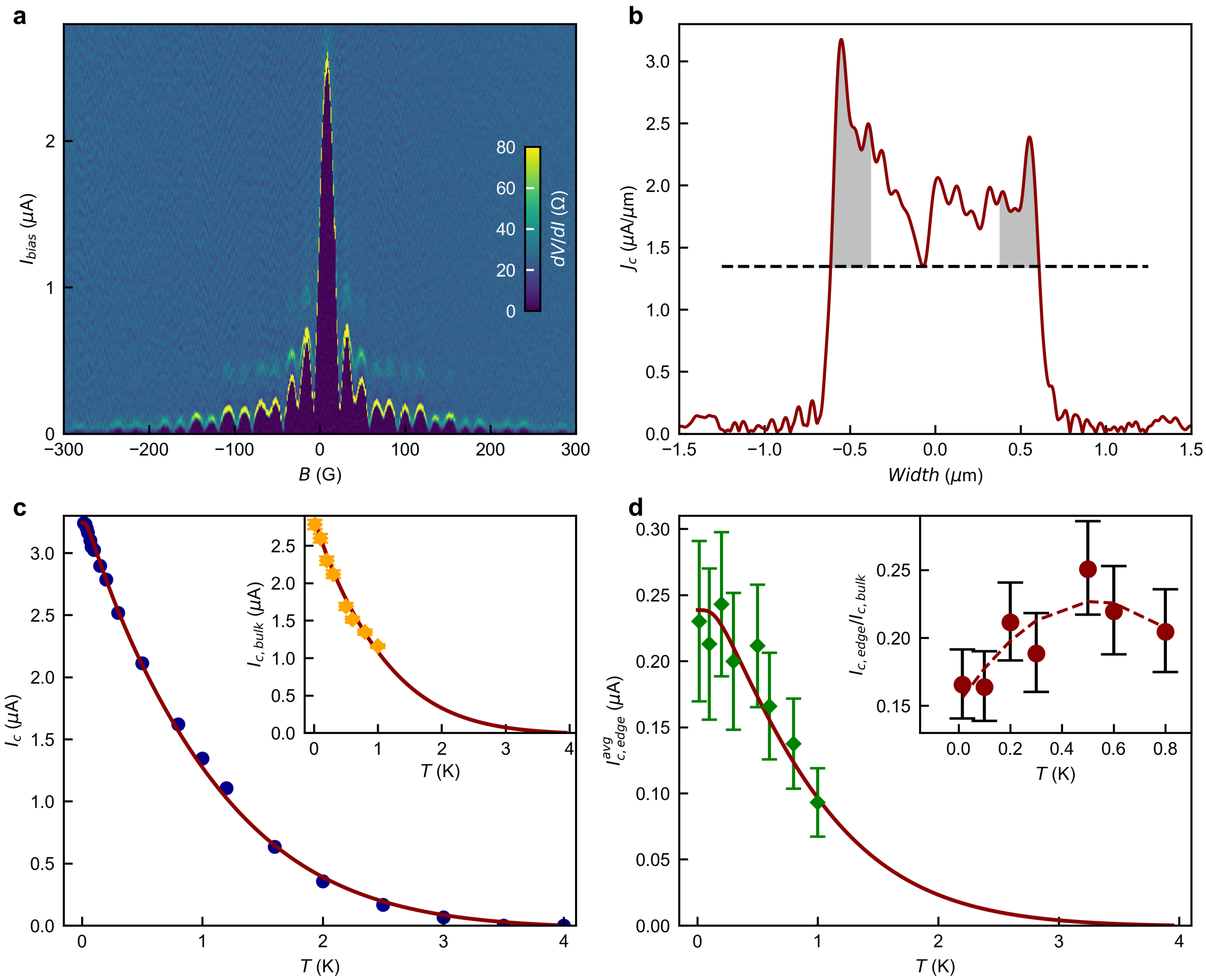

Fig S8 | **a**, I_cB map at 300 mK for 800 nm junction. The colormap represents the differential resistance dV/dI of the device with $R_N = 25\ \Omega$. **b**, Extracted $J_c(x)$ profile across the junction width showing enhanced density at edges typical for a junction featuring edge states. Edge states width of 266 nm has been estimated from our simulation. Gray shaded area highlights the edge-propagated I_c, which we estimate $I_{c,\ left\ edge} = 267.66$ nA and $I_{c,\ right\ edge} = 131.86$ nA. Dashed black line represents the minimum value of the bulk background contribution. **c**, Temperature dependence of total (both edges + bulk) I_c for this device. Experimental I_cT (darkblue dots) can be explained clearly with the Eilenberger theory (solid dark red). The estimated fitting parameters are $D = 0.9998$, $\xi = 260$ nm, $T_c = 4$ K and $R_N = 227\ \Omega$. Inset shows the temperature dependence of extracted $I_{c,\ bulk}$ from (**b**) which has been fitted with Eilenberger theory with fit parameters $D = 0.9998$, $\xi = 260$ nm, $T_c = 4$ K and $R_N = 266.8\ \Omega$. Error bars originate from the standard deviation between experimental and numerically calculated $J_c(x)$ pattern. **d**, Extracted average $I^{avg}_{c,\ edge}$ given by $(I_{c,\ left\ edge} + I_{c,\ right\ edge})/2$ variation with temperature (green diamonds). The Eilenberger theory fit parameters are $D = 0.99$, $\xi = 200$ nm, $T_c = 4$ K and $R_N = 2070.8\ \Omega$. Average number of channels per edge are 6 modes. Inset shows the $I_{c,\ edge}/I_{c,\ bulk}$ ratio ($I_{c,\ edge} = I_{c,\ left\ edge} + I_{c,\ right\ edge}$) for the 800 nm junction showing a similar increasing trend with temperature as seen for device F2_600 nm (Fig. 3d, main text). The dashed curve is guide-to-the-eye.

5 Supercurrent characteristics of F2_1000 nm Josephson junction

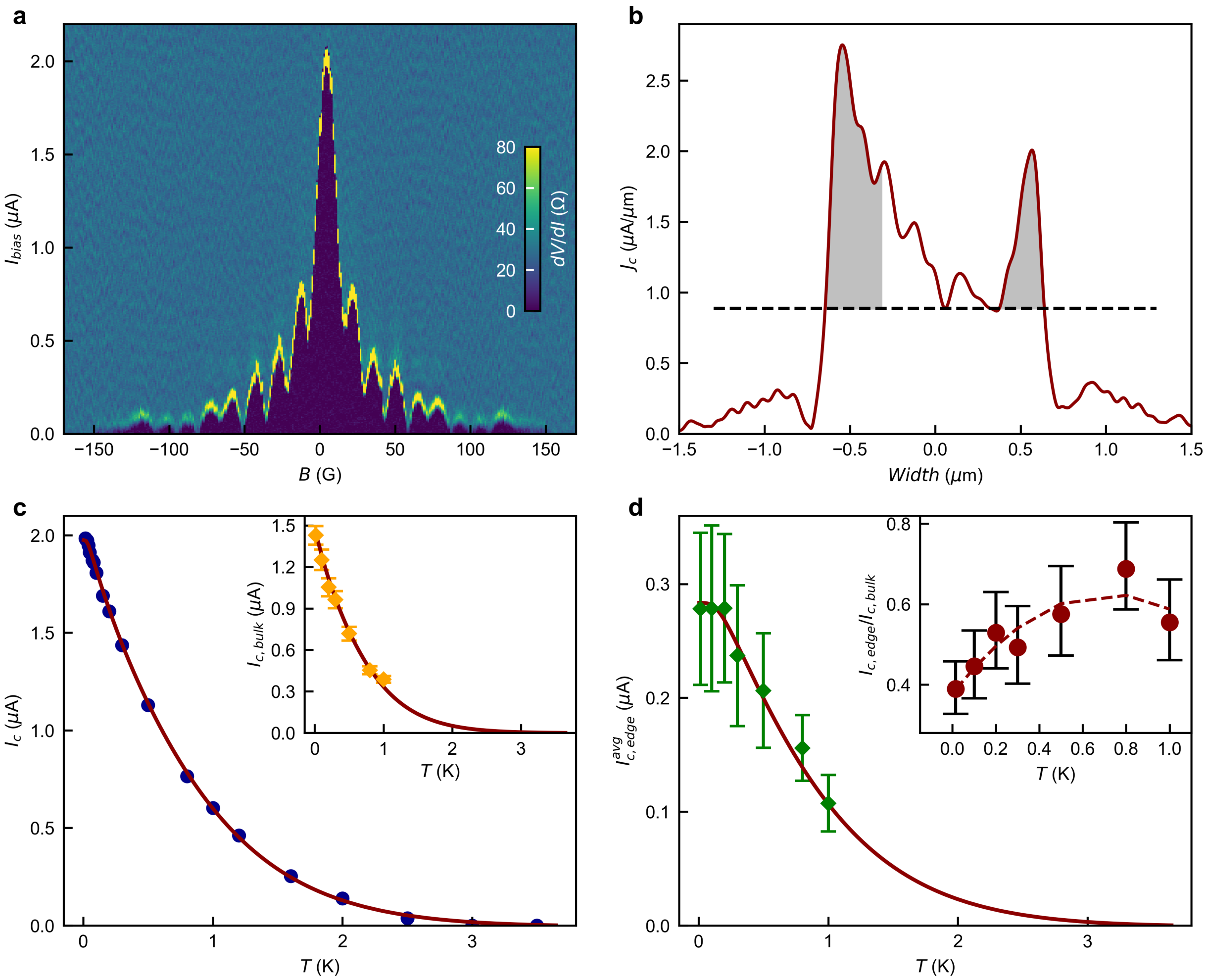

Fig S9 | **a**, I_cB map at 15 mK for 1000 nm junction. The colormap represents the differential resistance dV/dI of the device. R_N is 30 Ω for this device. **b**, Extracted $J_c(x)$ profile across the junction width showing enhanced density at edges typical for a junction featuring edge states. Edge states width of 381 nm has been estimated from our simulation. Gray shaded area highlights the edge-propagated I_c, which we estimate to be $I_{c,\ left\ edge} = 403.41$ nA and $I_{c,\ right\ edge} = 152.83$ nA. Dashed black line represents the minimum value of the bulk background contribution. **c**, Temperature dependence of total (both edges + bulk) I_c for this device. Experimental I_cT (dark blue dots) can be explained clearly with the Eilenberger theory (solid dark red). The estimated fitting parameters are $D = 0.9998$, $\xi =$ 260 nm, $T_c = 3.7$ K and $R_N = 302.6$ Ω. Inset shows the temperature dependence of extracted $I_{c,\ bulk}$ from (**b**) which has been fitted with Eilenberger theory with fit parameters $D = 0.9998$, $\xi = 200$ nm, $T_c = 3.7$ K and $R_N = 354.4$ Ω. Error bars originate from the standard deviation between experimental and numerically calculated I_cB pattern. **d**, Extracted average $I^{avg}_{c,\ edge}$ given by $(I_{c,\ left\ edge} + I_{c,\ right\ edge})/2$ variation with temperature (green diamonds). The Eilenberger theory fit parameters are $D = 0.99$, $\xi = 260$ nm, $T_c = 3.7$ K and $R_N = 1652.6$ Ω. Average number of channels per edge are 8 modes. Inset shows the $I_{c,\ edge}/I_{c,\ bulk}$ ratio ($I_{c,\ edge} = I_{c,\ left\ edge} + I_{c,\ right\ edge}$) for the 1000 nm junction showing a similar increasing trend with temperature as seen for the other two junctions on this flake. The dashed curve is guide-to-the-eye.

6 RF dependence of missing odd Shapiro steps in F2_600 nm junction

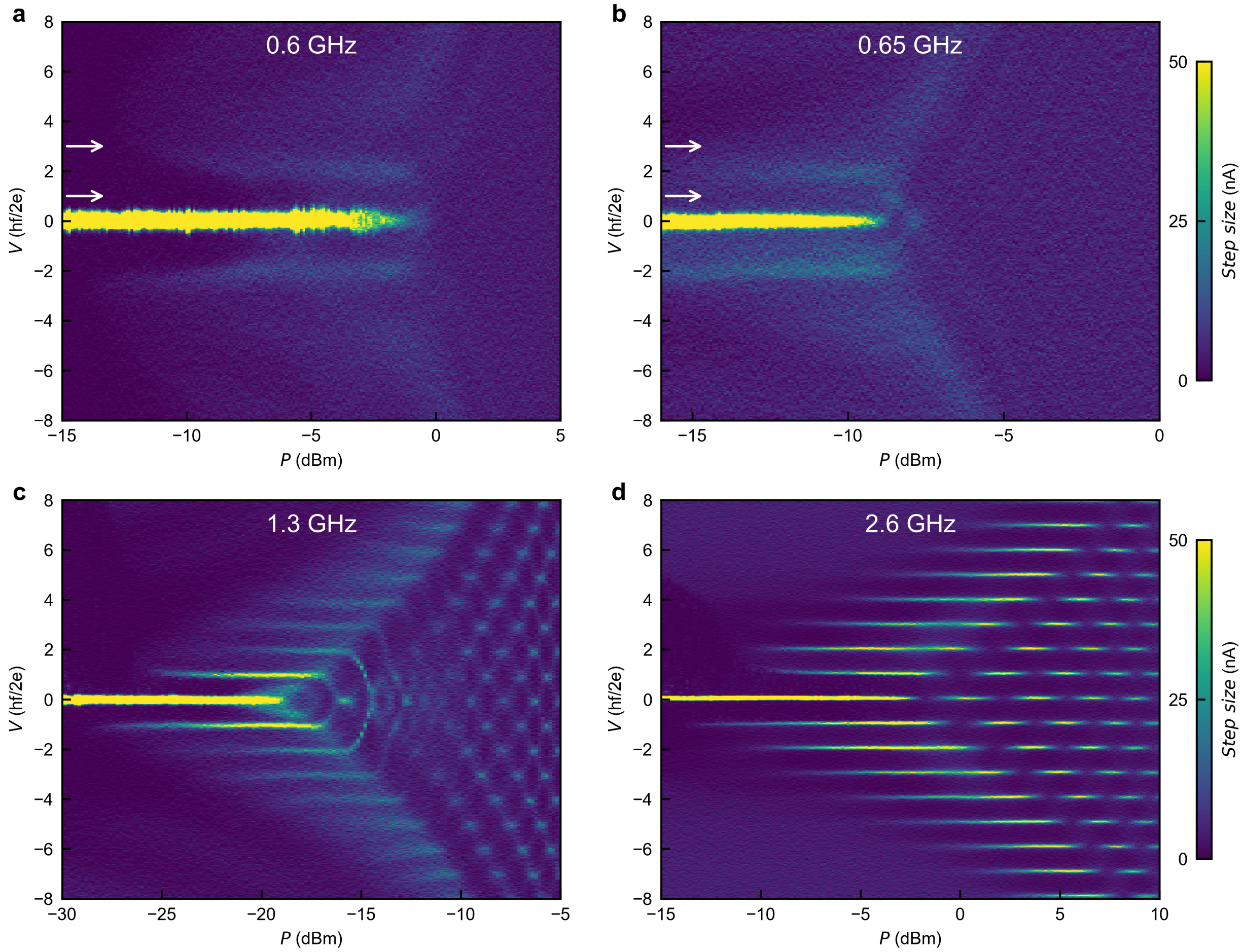

Fig S10 | Re-appearance of the odd Shapiro steps at higher frequency for device F2_600 nm. White arrows mark the positions of the missing first and third odd integer steps at $f =$ (**a**) 0.6 GHz and (**b**) 0.65 GHz. Figure 3a of main text shows the missing steps at 0.9 GHz. (**a**) and (**b**) share the same voltage bin size and the colorscale. All the Shapiro steps are present at higher RF, $f =$ (**c**) 1.3 GHz and (**d**) 2.6 GHz. (**c**) and (**d**) share the same voltage bin size and the colorscale.

7 Temperature dependence of missing odd Shapiro steps in F2_600 nm junction

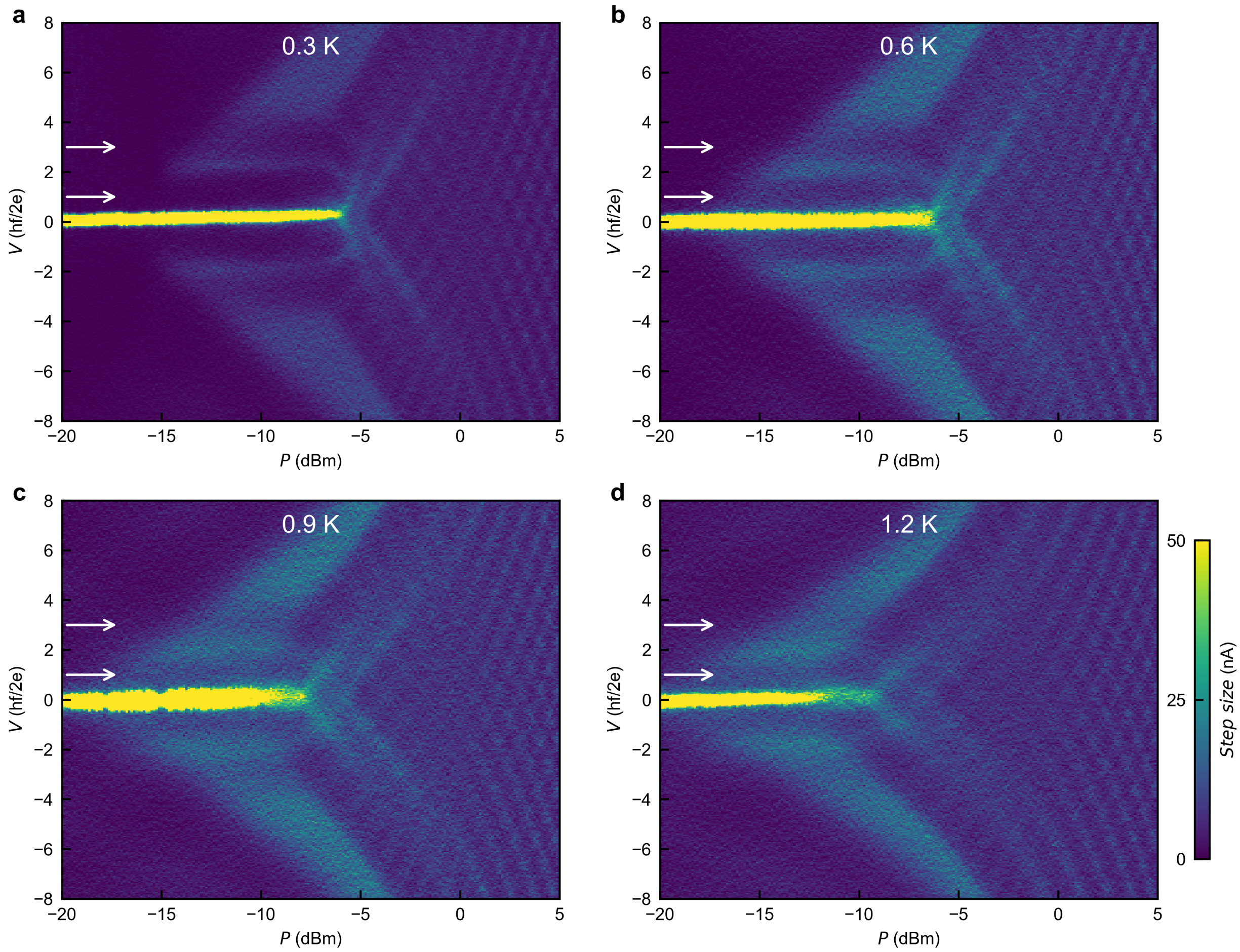

Fig S11 | Robustness of the missing Shapiro steps with increasing temperature for device F2_600 nm. Same voltage bin size has been used for all the temperatures. White arrows mark the positions of the missing first and third odd integer steps at $T =$ (**a**) 0.3 K, (**b**) 0.6 K, (**c**) 0.9 K and (**d**) 1.2 K. Thermal smearing of the quantized voltage steps can be seen with increasing temperature.

8 Missing odd Shapiro steps in F2_800 nm junction

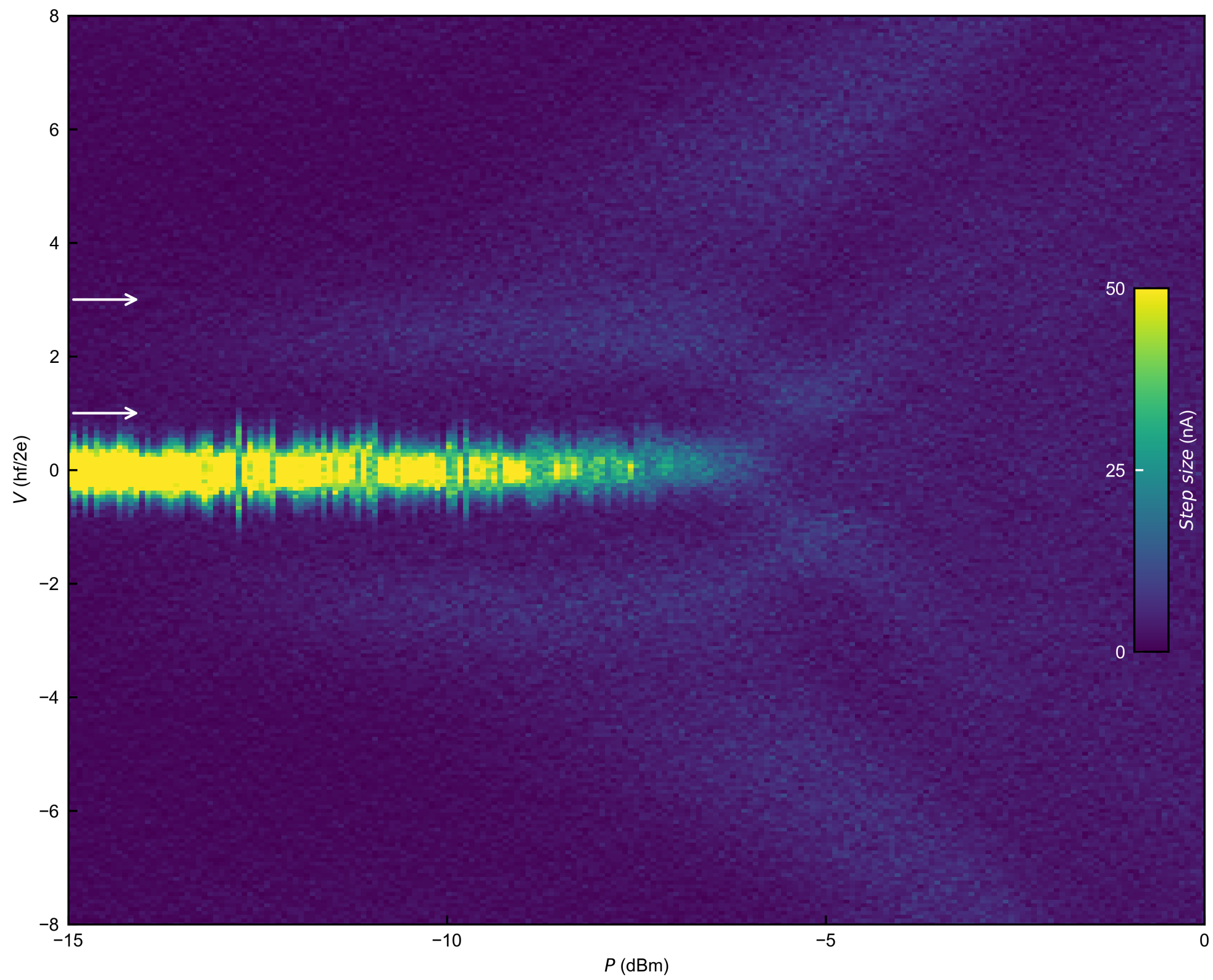

Fig S12 | **a**, Missing odd steps ($n = 1,\ 3$) for the F2_800 nm device under excitation with low RF irradiation $f = 0.65$ GHz at 70 mK. White arrows mark the positions of the missing Shapiro steps.

9 Supercurrent characteristics of F1 bulk-edge Josephson junction device

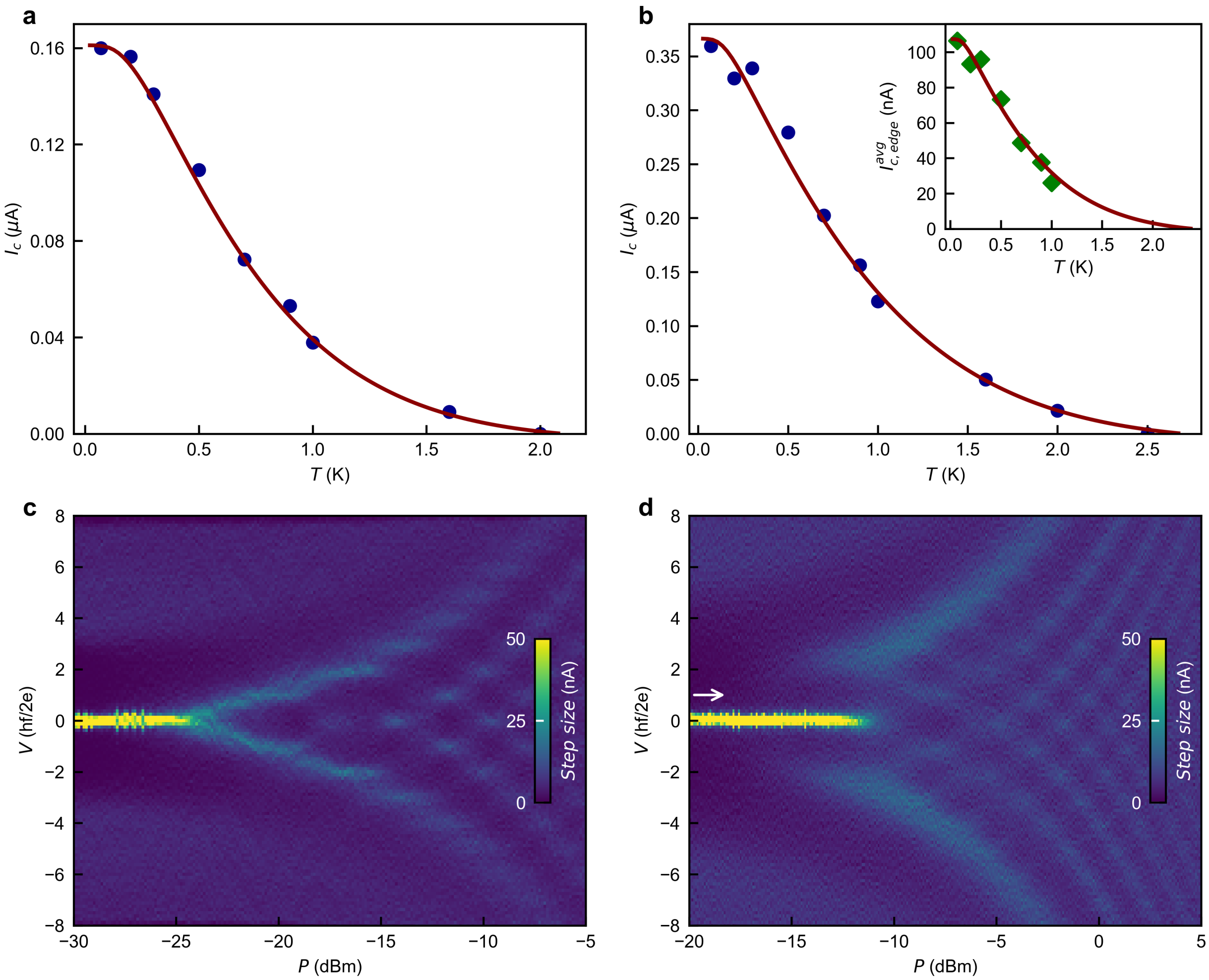

Fig S13 | **a**, Temperature dependence of total I_c for device F1_bulk JJ with $L = 800$ nm. Experimental data points (dark blue) can be fitted with Eilenberger theory (solid dark red). The estimated fitting parameters are $D = 0.95$, $\xi = 300$ nm, $T_c = 2.1$ K and $R_N = 1530\ \Omega$. **b**, Temperature dependence of total I_c for device F1_edge JJ with $L = 800$ nm can also be fitted with Eilenberger theory. The estimated fitting parameters are $D = 0.99$, $\xi = 340$ nm, $T_c = 2.7$ K and $R_N = 1234.5\ \Omega$. Inset shows the temperature dependence of the extracted I_c for the edge modes (green diamonds) given by $(I_{c,\,left\ edge} + I_{c,\,right\ edge})/2$. The Eilenberger theory fit parameters are $D = 0.99$, $\xi = 340$ nm, $T_c = 2.4$ K and $R_N = 3743.3\ \Omega$. **c, d**, RF measurements showing the Shapiro steps in F1_bulk-edge JJ devices at 70 mK. While all the integer steps are observed for the bulk JJ (**c**) at 0.85 GHz, there is a missing odd $n = 1$ step for the edge JJ (**d**) at 0.8 GHz marked by white arrow. We did not observe missing odd steps for the bulk JJ at any other frequency. This is a strong evidence of topological hinge modes in this material.

10 Monotonically decaying $I_c B$ for different bulk Josephson junctions

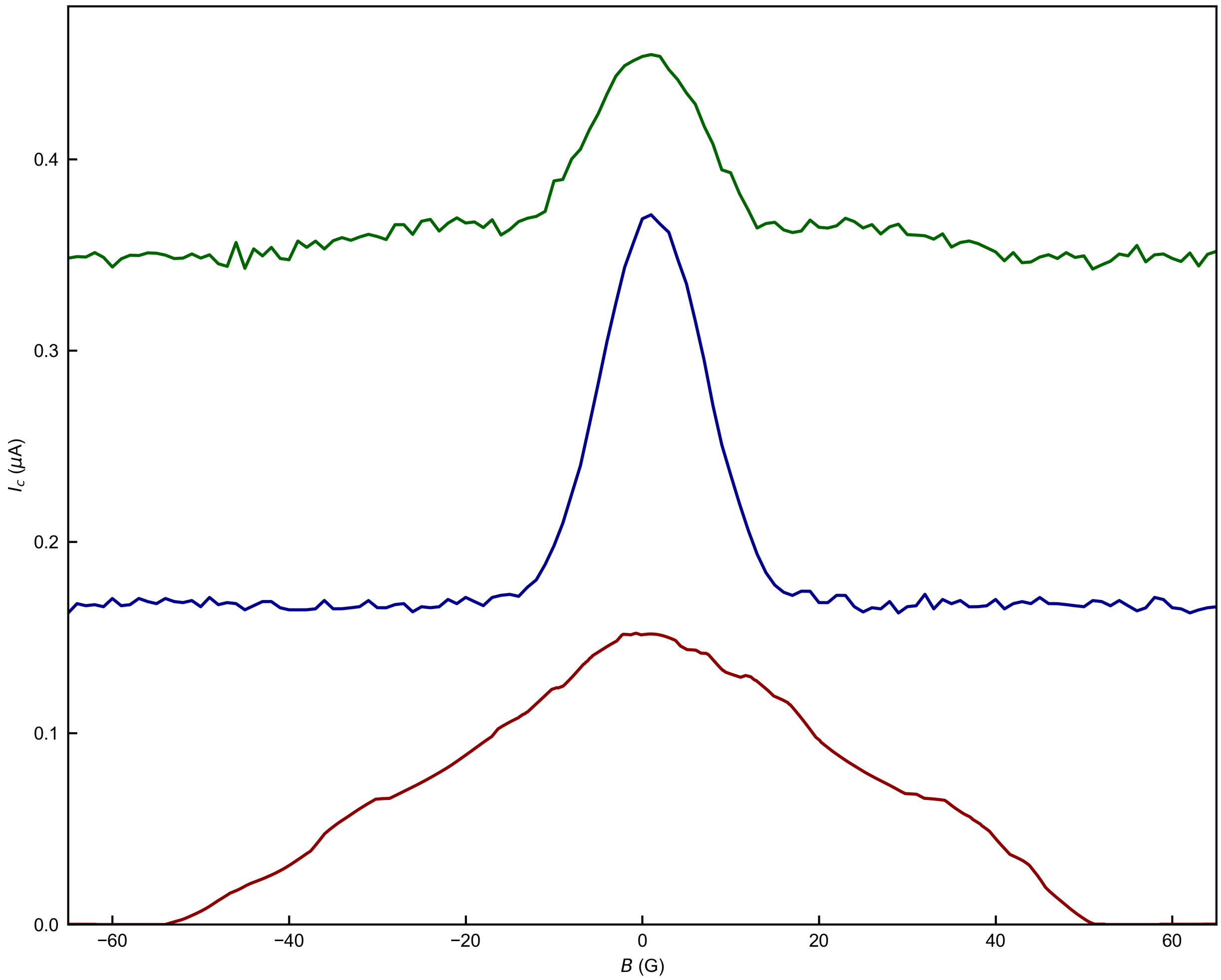

Fig S14 | Monotonically decaying $I_c B$ pattern is observed for three different bulk JJs on separate flakes (curves overlayed for clarity). Dimensions of the bulk JJ device are as follows: L = 600 nm, W = 1.2 μm, t = 150 nm for green curve, L = 800 nm, W = 1.2 μm, t = 150 nm for blue curve and L = 800 nm, W = 1 μm, t = 200 nm for red curve.

11 Surface morphology of the flake F2 probed with atomic force microscopy

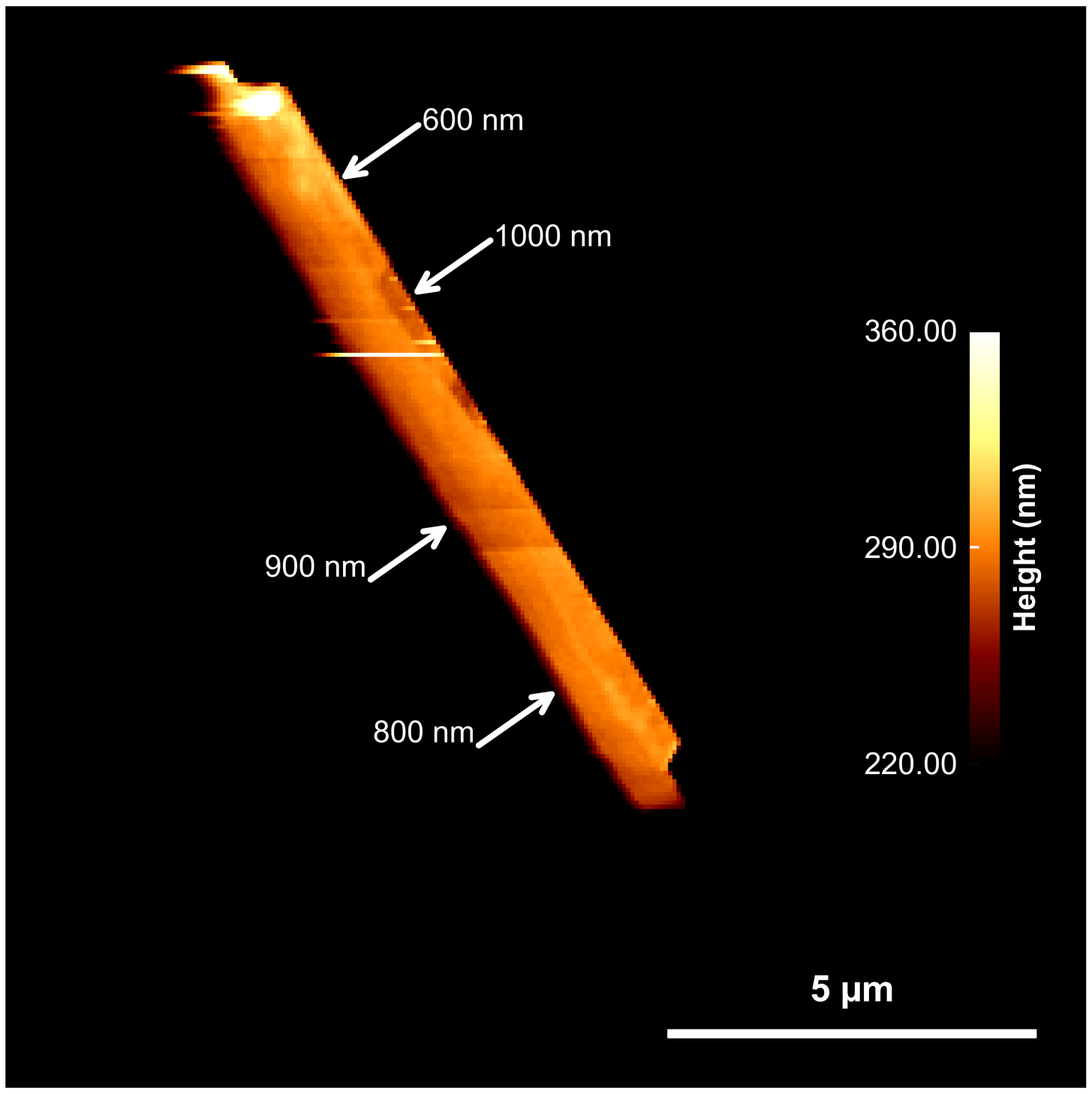

Fig S15 | Atomic force microscopy (AFM) image of flake F2 highlighting the surface morphology and positions of the different junctions marked by white arrows. The thickness ($\sim$ 250 nm) of the flake is uniform throughout it's entire length. The colorscale is chosen in a way that it increases the contrast and provides better resolved surface morphology.

Figure S15 shows the AFM height sensor image for flake F2 with position of the four different junctions fabricated on this flake marked by white arrows (see Fig. 2a for device image). An additional large step ($\sim$ 15 nm high) on one of the edges can be seen for the 1000 nm junction. Interestingly, in order to emulate the I_cB and $J_c(x)$ of this junction, we had to consider this step on one edge in our simulation too. A small bump can be seen at the center of flake F2 where 800 nm junction is placed, which also renders a small bump in its $J_c(x)$ distribution around the middle of the junction width (Fig. S8b). This shows the sensitivity of Dynes and Fulton Fourier techniques to the real surface morphology of the junction.

12 SEM characterization

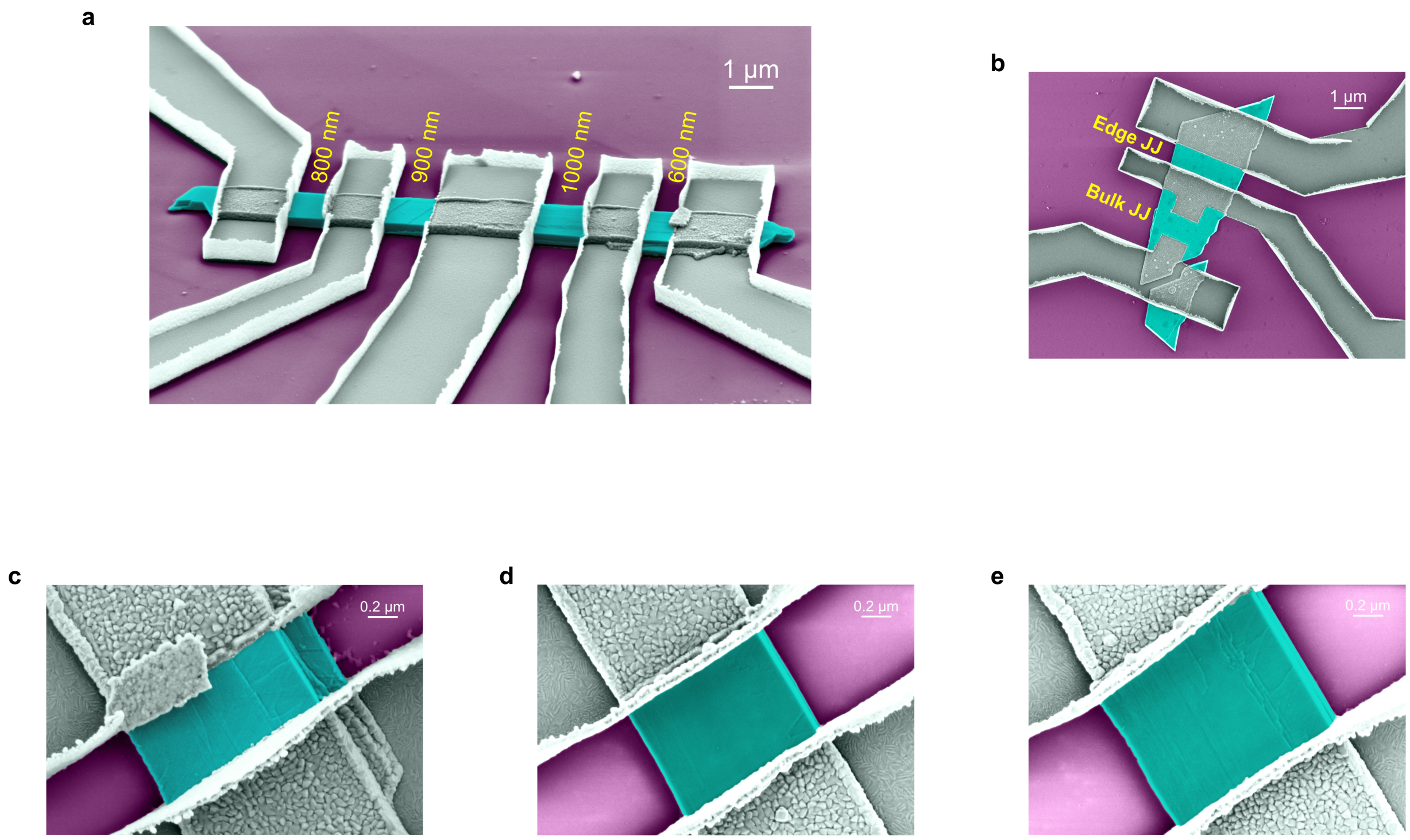

Fig S16 | **a**, Cross-sectional SEM image of flake F2 highlighting the different junctions. **b**, SEM image of flake F1 showing the bulk and edge JJs both 800 nm long. **c-e**, SEM images of 600, 800 and 1000 nm junctions in **a**.

13 Q_{12} calculation in RF Shapiro steps measurements

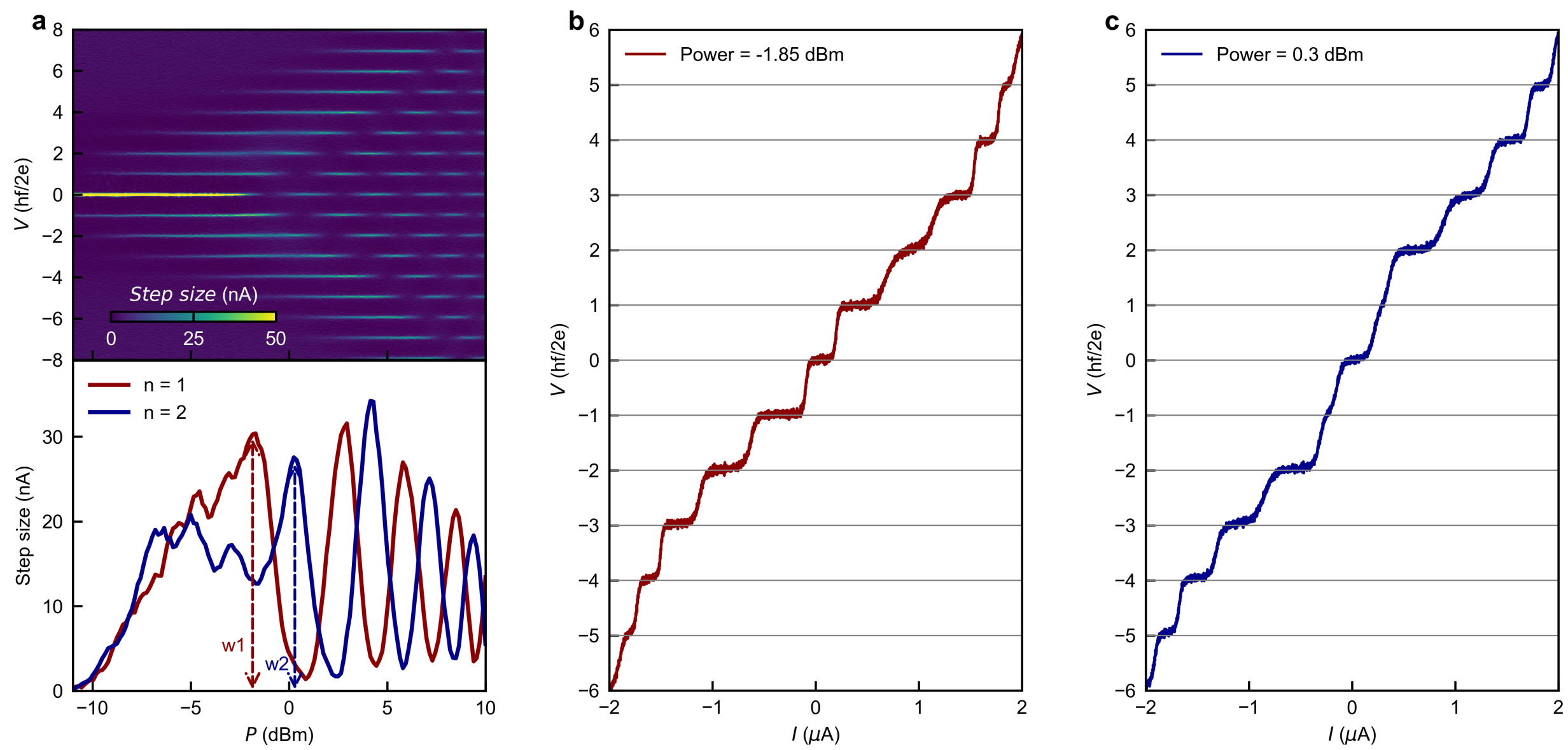

Fig S17 | **a**, Colormap showing the Shapiro steps for device F2_600 nm under 2.65 GHz RF irradiation at 70mK. Bottom panel are the line-cuts from top panel at $n = 1$ and 2 depicting the power dependence of their step sizes. $w1$ and $w2$ mark the step size width of $n = 1$ and 2 as the maximum value of first lobe in the power dependence of step size. The ratio $Q_{12} = w1/w2$, thus provides information about the suppression of $n = 1$ step. **b,c**, IV curves (raw data) with voltage normalized to $\frac{hf}{2e}$ at powers of -1.85 dBm ($w1$) and 0.3 dBm ($w2$) showing the quantized step sizes for reference.

14 Supercurrent characteristics of a shorter ($L = 350$ nm) Josephson junction

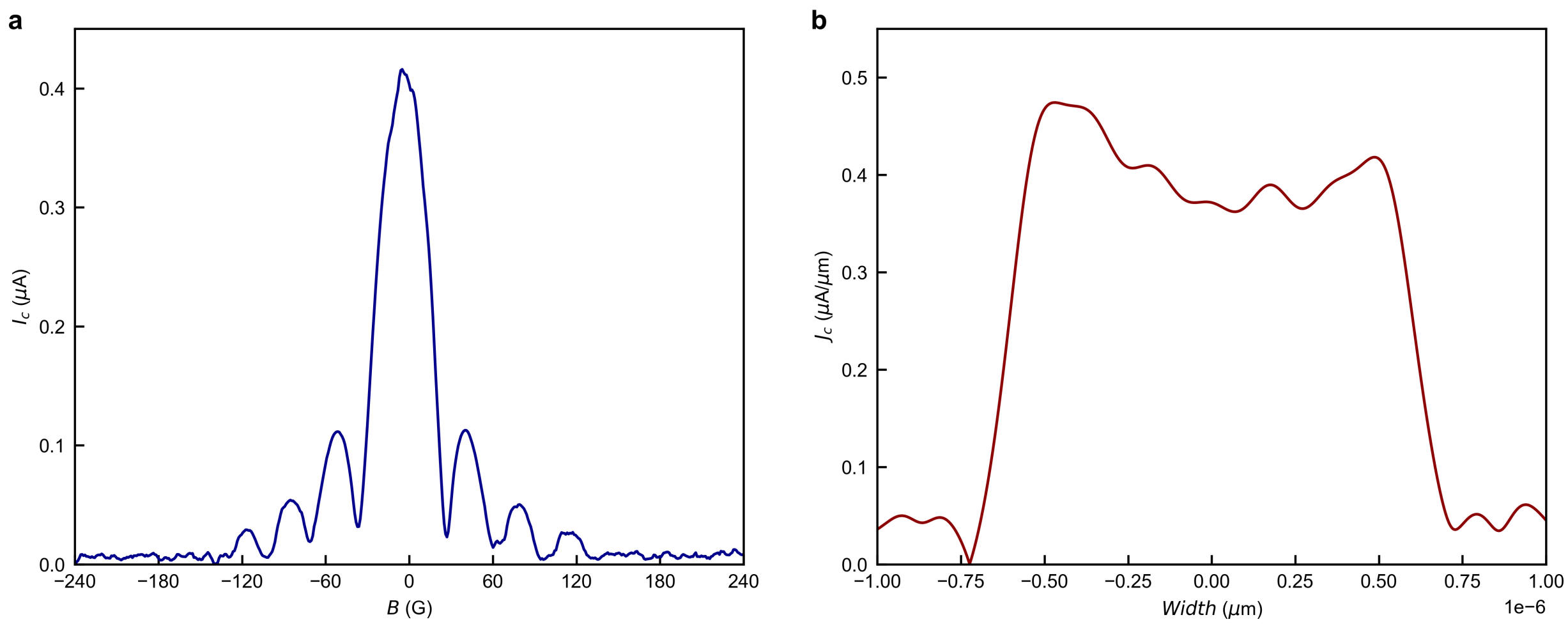

Fig S18 | **a**, Shorter junctions allow both bulk and edge channels to survive ballistically, producing the standard single-slit Fraunhofer interference. **b**, Extracted $J_c(x)$ distribution of across this junction width shows bulk-dominated conduction. In contrast, the bulk coherence decays for longer junctions, leaving only robust edge channels that dominate and yield SQUID-like patterns as shown in previous devices. This transition reflects a length-dependent crossover between the bulk and edge transport channels.

15 Table: Summary of device parameters and properties

Device **#**	$t\ (nm)$	$L\ (nm)$	$W\ (\mu m)$	$I_cR_N\ (\mu V)$	I_cB	RF
BS04_1	50	300	0.3	16	Monotonic decay	Not measured
BS04_2	110	300	0.7	40	Typical Fraunhofer	Not measured
BS07_CF1-bulk	150	600	1.2	4.7	Monotonic decay	Not measured
BS07_BF8-bulk	150	800	1.2	14.1	Monotonic decay	Not measured
BS06_F5	170	1000	1.8	7.7	SQUID-like	All steps present
BS06_F12	175	350	1	25.7	Typical Fraunhofer	All steps present
BS07_F1-edge	200	800	2.7	9.7	SQUID-like	Missing $n = 1$
BS07_F1-bulk	200	800	1	7.2	Monotonic decay	All steps present
BS07_F2	250	600	1.4	82.5	SQUID-like	Missing $n = 1,\ 3$
BS07_F2	250	800	1.3	77.5	SQUID-like	Missing $n = 1,\ 3$
BS07_F2	250	900	1.4	50.4	SQUID-like	Not measured
BS07_F2	250	1000	1.4	56.1	SQUID-like	All steps present

Table S1 | Summary of device parameters and properties. (R_N is from experimental dV/dI)

16 Brillouin zone schematic of $Bi_{0.97}Sb_{0.03}$ in the momentum space

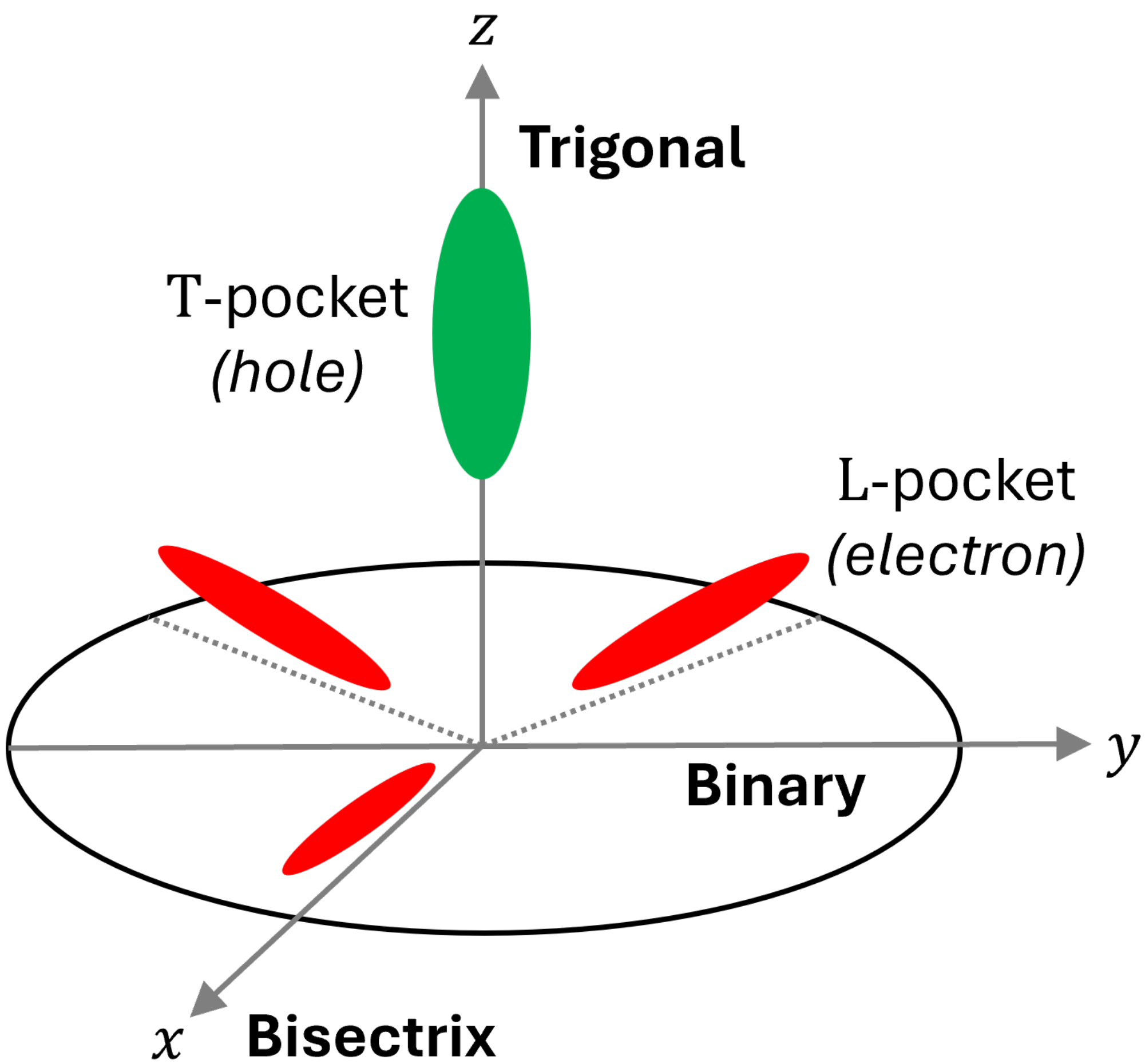

Fig S19 | Brillouin zone schematic of $Bi_{0.97}Sb_{0.03}$ in the momentum space with one hole pocket at the T-point and three electron pockets at the L-point. The Nb electrodes are aligned in a way that current bias is always along the bisectrix axis of the $Bi_{0.97}Sb_{0.03}$ flake.

17 Energy-dispersive X-ray spectroscopy (EDS) of the $Bi_{0.97}Sb_{0.03}$ crystal

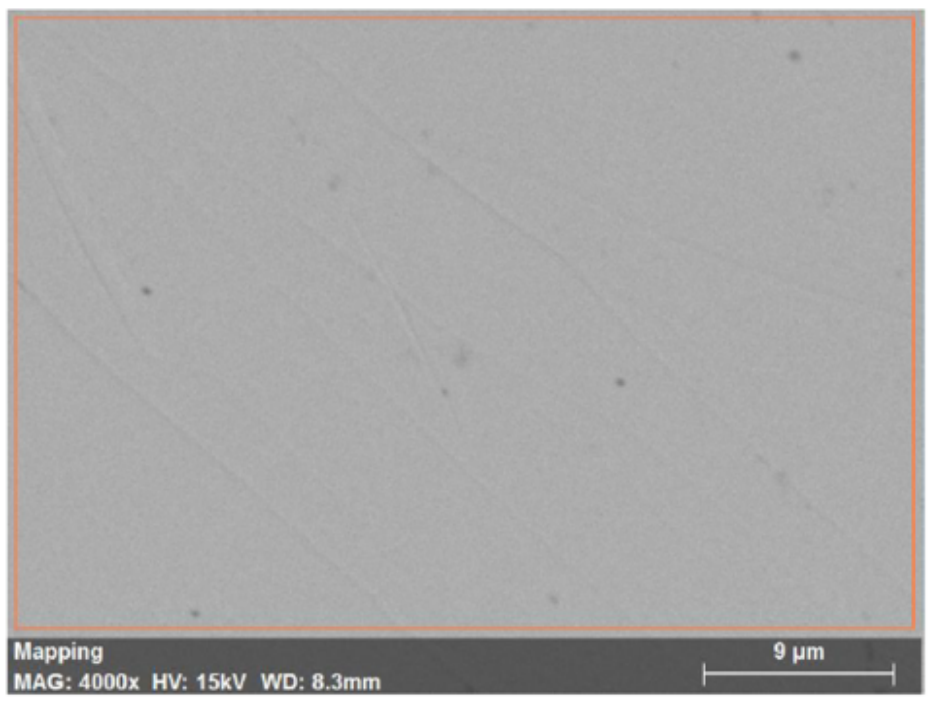

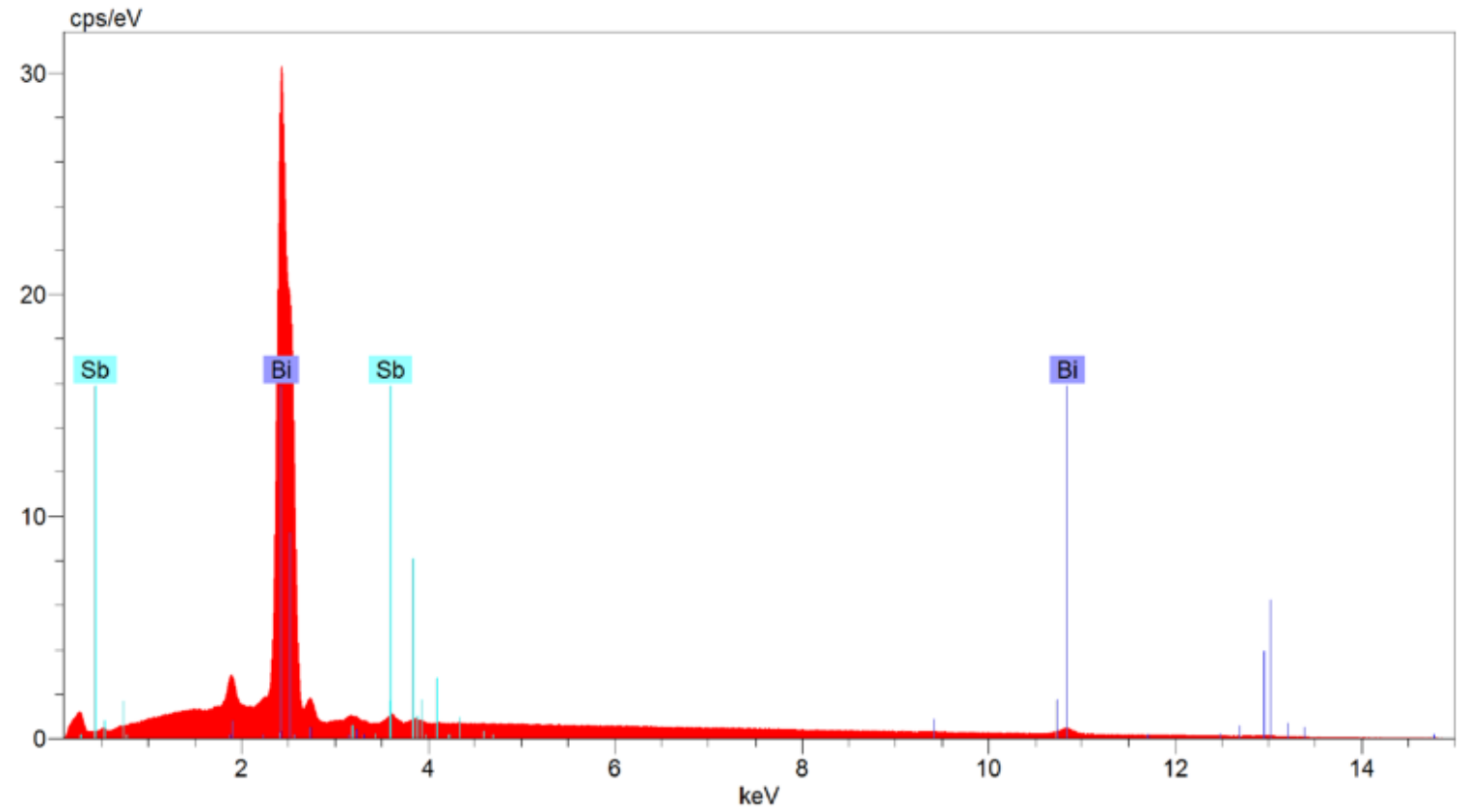

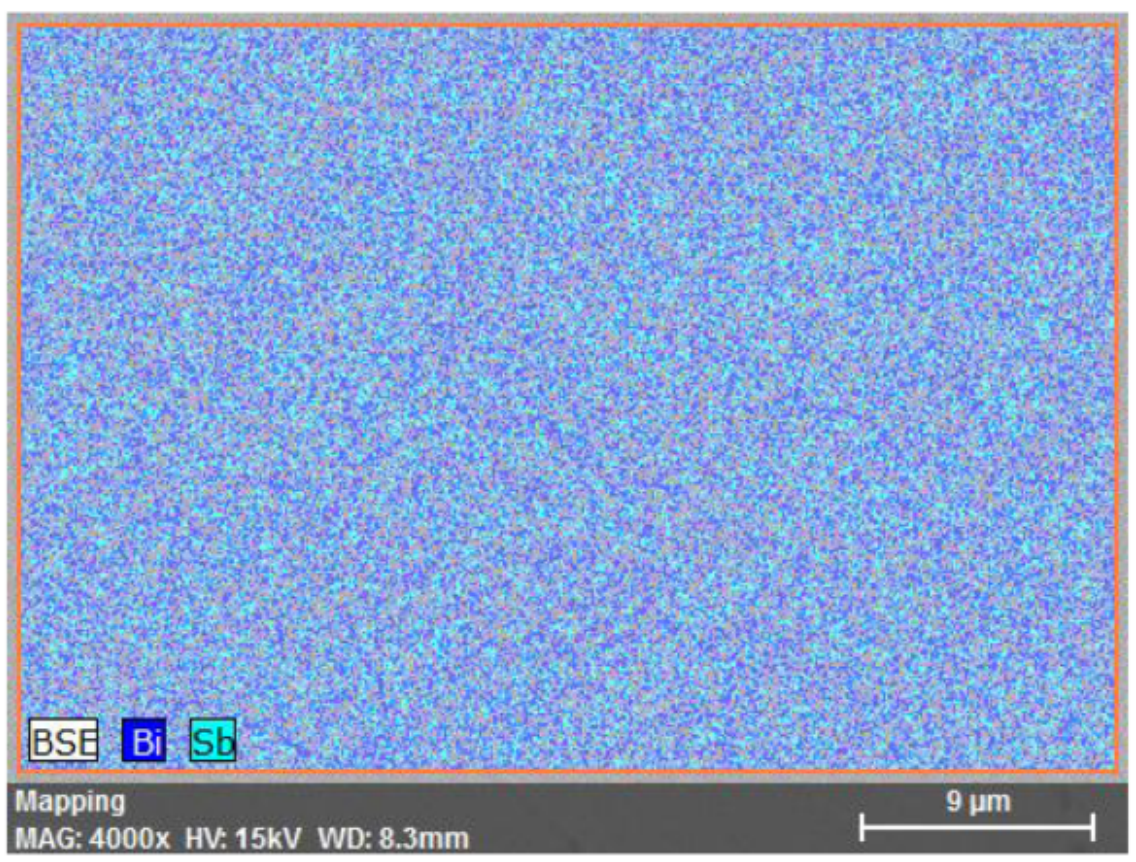

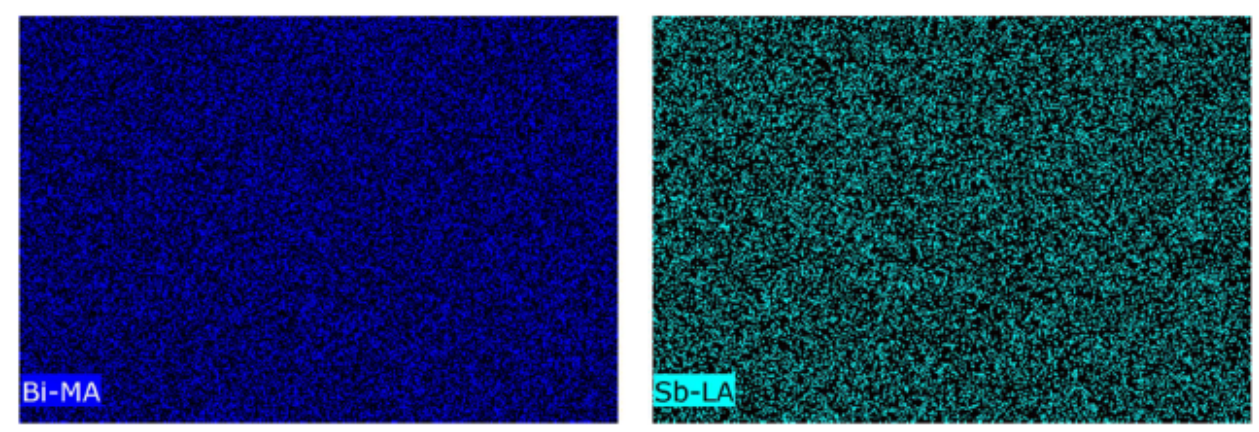

El	AN	Series	unn. C [wt.%]	norm. C [wt.%]	Atom. C [at.%]	Error [%]
Bi	83	M-series	92.68	98.24	97.02	3.2
Sb	51	L-series	1.66	1.76	2.98	0.1
		Total:	94.34	100.00	100.00	

Fig S20 | EDS spectra at magnification = 4000x and high voltage = 15 kV, table shows the atomic concentrations in %. EDS data was taken at multiple random locations and at different magnifications, which consistently showed a uniform distribution of bismuth and antimony across the scanned area alongwith atomic concentrations of $\sim 97\%$ for Bi and $\sim 3\%$ for Sb, respectively. The characteristic X-ray energies for Bi and Sb are in good agreement with the reported literature values.[6,7]

References

1 Chen, A. Q. *et al.* Finite momentum Cooper pairing in three-dimensional topological insulator Josephson junctions. *Nature Communications 2018 9:1* **9**, 1–9 (2018).

2 Liu, Y. & Allen, R. E. Electronic structure of the semimetals Bi and Sb. *PHYSICAL REVIEW B* **8**, 15 (1995).

3 Groth, C. W., Wimmer, M., Akhmerov, A. R. & Waintal, X. Kwant: a software package for quantum transport. *New Journal of Physics* **16**, 063065 (2014). URL `http://dx.doi.org/10.1088/1367-2630/16/6/063065`.

4 Murakami, S. Quantum spin hall effect and enhanced magnetic response by spin-orbit coupling. *Physical Review Letters* **97**, 1–4 (2006). `0607001`.

5 Dynes, R. C. & Fulton, T. A. Supercurrent density distribution in josephson junctions. *Physical Review B* **3**, 3015–3023 (1971). URL `http://dx.doi.org/10.1103/PhysRevB.3.3015`.

6 Dao, N. N. *et al.* Low-temperature synthesis and investigations on photocatalytic activity of nanoparticles bifeo3 for methylene blue and methylene orange degradation and some toxic organic compounds. *Advances in Natural Sciences: Nanoscience and Nanotechnology* **7**, 045003 (2016). URL `http://dx.doi.org/10.1088/2043-6262/7/4/045003`.

7 Chihi, A. Annealing effect on sb2s3/c-si structure for photovoltaic applications. *Applied Physics A* **130** (2024). URL `http://dx.doi.org/10.1007/s00339-024-07692-4`.
